# Systematic Identification of *Plasmodium Falciparum* Sporozoite Membrane Protein Interactions Reveals an Essential Role for the p24 Complex in Host Infection

**DOI:** 10.1074/mcp.RA120.002432

**Published:** 2021-01-27

**Authors:** Julia Knöckel, Kirsten Dundas, Annie S.P. Yang, Francis Galaway, Tom Metcalf, Geert-Jan van Gemert, Robert W. Sauerwein, Julian C. Rayner, Oliver Billker, Gavin J. Wright

**Affiliations:** 1 Cell Surface Signalling Laboratory, Wellcome Sanger Institute, Cambridge, United Kingdom; 2 Malaria Programme, Wellcome Sanger Institute, Cambridge, United Kingdom; 3 Radboudumc Center for Infectious Diseases, Department of Medical Microbiology, Radboud University Medical Center, Nijmegen, The Netherlands; 4 The Laboratory for Molecular Infection Medicine Sweden (MIMS) and Department of Molecular Biology, Umeå University, Umeå, Sweden; 5 Department of Biology, Hull York Medical School, York Biomedical Research Institute, University of York, York, United Kingdom

**Keywords:** AVEXIS, avidity-based extracellular interaction screen, CSP, circumsporozoite protein, GPI, glycosylphosphatidylinositol, HBS, hepes-buffered saline, HEK, human embryonic kidney, IVIS, *in vivo* imaging system, MSP, merozoite surface protein, PIESP15, parasite-infected erythrocyte surface protein 15, SPR, surface plasmon resonance, TRAP, thrombospondin-related anonymous protein

## Abstract

Sporozoites are a motile form of malaria-causing *Plasmodium falciparum* parasites that migrate from the site of transmission in the dermis through the bloodstream to invade hepatocytes. Sporozoites interact with many cells within the host, but the molecular identity of these interactions and their role in the pathology of malaria is poorly understood. Parasite proteins that are secreted and embedded within membranes are known to be important for these interactions, but our understanding of how they interact with each other to form functional complexes is largely unknown. Here, we compile a library of recombinant proteins representing the repertoire of cell surface and secreted proteins from the *P. falciparum* sporozoite and use an assay designed to detect extracellular interactions to systematically identify complexes. We identify three protein complexes including an interaction between two components of the p24 complex that is involved in the trafficking of glycosylphosphatidylinositol-anchored proteins through the secretory pathway. *Plasmodium* parasites lacking either gene are strongly inhibited in the establishment of liver-stage infections. These findings reveal an important role for the p24 complex in malaria pathogenesis and show that the library of recombinant proteins represents a valuable resource to investigate *P. falciparum* sporozoite biology.

## Highlights

• A library of *Plasmodium falciparum* sporozoite membrane and secreted proteins.
• Systematic extracellular interaction screening identifies novel protein complexes.
• Interactions are conserved across *Plasmodium* species.
• Essential role for genes encoding p24 complex proteins in liver-stage infection.

## Graphical Abstract

**Figure:**
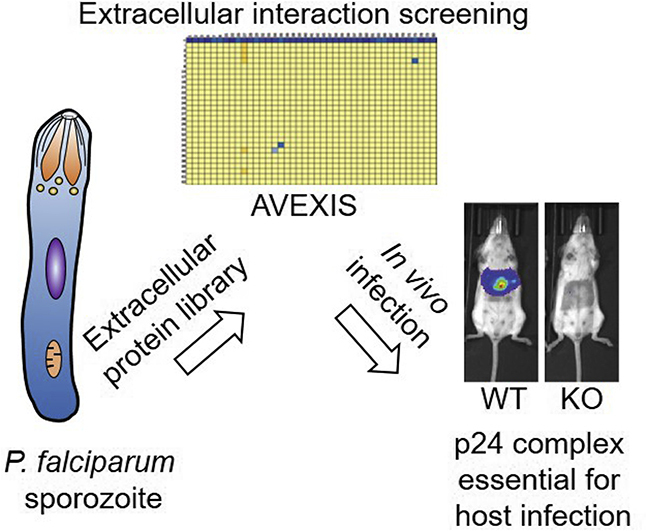

## In Brief

Malaria remains one of the world's most deadly infectious diseases, and host infection is initiated when the sporozoite form of the *P. falciparum* parasite is transmitted by the bite of an infected mosquito. Here, we compile a library of soluble recombinant sporozoite cell surface and secreted proteins and test them for direct interactions using an extracellular protein interaction assay. We identify three conserved complexes and show that one, the p24 complex, is essential for host liver-stage infection.

Malaria is a deadly mosquito-borne infectious disease caused by unicellular *Plasmodium* parasites and despite intensive control efforts is still responsible for almost half a million deaths annually (1). Critical to mosquito–human transmission is the sporozoite: a motile stage that develops within oocysts located on the midgut of infected mosquitoes before invading the mosquito salivary glands and being transmitted during a subsequent blood meal. Once deposited in the dermis, sporozoites disperse in apparently random directions before entering the circulation and eventually enter the liver where they invade hepatocytes and continue their development (2). Targeting the sporozoite is an attractive choice for vaccine development because sporozoites are extracellular and therefore directly exposed to the host humoral immune system, this stage of the infection is asymptomatic, and sporozoites are few in number since only tens to hundreds are transmitted (3, 4, 5). Of importance, malaria vaccines based on attenuated whole sporozoites, as well as a subunit vaccine incorporating components of the major sporozoite surface protein CSP, have been shown to be effective in human trials, although protection was typically short-lived (6).

Proteins that are displayed on the surface of the parasite or released from intracellular secretory organelles such as the rhoptries and micronemes are considered good subunit vaccine targets because they are directly accessible to host antibodies. The main functions of these proteins are likely to be the subversion of the immune response or the interaction with host molecules to regulate different stages of infection, for example, to power parasite motility or bind specific receptors that control the tropism of host cell invasion. Because the blood stages of *Plasmodium falciparum* can be cultured *in vitro*, we have a comparatively good understanding of which parasite proteins are displayed on the surface of the merozoite and how they interact to form functional complexes. For example, the major merozoite surface protein MSP1 is found in a protein complex with MSP7 and MSP6 (7, 8) and the secreted P41 protein is tethered to the surface of the merozoite by the glycosylphosphatidylinositol (GPI)-anchored P12 protein (9). Parasite protein complexes that are known to be essential for *P. falciparum* erythrocyte invasion include the interactions between AMA1 and RON2 (10) and more recently those proteins that directly bind RH5 including cysteine-rich protective antigen (11), RH5 interacting protein (12), and P113 (13). Mechanistically, vaccine-elicited antibodies that target the components of these protein complexes on the parasite surface may neutralize blood-stage parasites by inhibiting their function or formation (14). In contrast to the blood stages, however, relatively little is known about the biochemical composition and organization of the sporozoite surface, although genetic targeting has revealed that several parasite surface proteins are required for normal sporozoite development and infection. For example, circumsporozoite protein (CSP) is required for the development of sporozoites in the oocyst (15, 16), TRAP family proteins (TRAP and TLP) are important for sporozoite motility and infection (17, 18), and members of the 6-cys family (P36 and P52) are important for host receptor usage in hepatocyte invasion (19). Although we have a good cellular description of how the sporozoite interacts with host cells (20), a molecular understanding of these behaviors is incomplete and yet could represent important new opportunities for pre-erythrocytic malaria vaccines.

Identification of interactions between membrane-embedded proteins is challenging because membrane proteins are both amphipathic, making them difficult to solubilize in solvents that retain their native conformation, and because interactions between them can be highly transient; together, these features make it difficult to confidently interpret data from commonly used methods such as biochemical purifications using native material (21). To address these issues, we have developed an approach called AVEXIS (for AVidity-based EXtracellular Interaction Screening) (22), which, together with a library of functionally active recombinant blood-stage *Plasmodium* proteins (23, 24), has permitted the biochemical identification and characterization of parasite protein complexes at the surface of the merozoite including the RH5 complex (13). Here, we use proteomic analyses of *Plasmodium* sporozoites to compile a recombinant protein library representing the cell surface receptor repertoire and secretome of the *P. falciparum* sporozoite. We use this protein library and the AVEXIS approach to identify interactions between sporozoite proteins and characterize their role in the pathogenesis of *Plasmodium* infections.

## Experimental Procedures

### Ethics Statement

Experiments involving animals were performed under UK Home Office governmental regulations (project license numbers PD3DA8D1F and P98FFE489) and European directive 2010/63/EU. Research was approved by the Sanger Institute Animal Welfare and Ethical Review Board.

### Design of the *Plasmodium* Sporozoite Protein Library

To obtain a catalog of proteins that are expressed by sporozoites, published proteomic data using mass spectrometry–based profiling of midgut and salivary gland sporozoites (25, 26, 27) were combined to create a list of unique proteins and protein expression constructs designed essentially as described (23); we additionally included several proteins expressed by ookinetes. Briefly, cell surface and secreted proteins were initially identified by the presence of a signal peptide and/or transmembrane domains using SignalP v4.0 (28) and TMHMM v2.0 (29) software. To define the ectodomain regions, signal sequences and transmembrane domains were removed and all potential N-linked glycosylation sites (N-X-S/T, where X is not proline) were systematically mutated by substituting the serine/threonine for alanine to prevent inappropriate glycosylation when expressed in mammalian cells. Sequences encoding the sporozoite protein ectodomains were codon optimized for expression in human cells and made by gene synthesis (GeneART). Four genes failed synthesis and three could not be subcloned into the mammalian expression plasmids, suggesting the insert was toxic in *Escherichia coli* (Table S1). All sequences were flanked with unique 5′ NotI and 3′ AscI restriction enzyme sites to allow inframe cloning in the bait and prey plasmids (30). Both expression plasmids contain a highly efficient mouse variable κ light chain signal peptide and a rat Cd4d3+4 epitope tag followed by either a peptide sequence allowing biotinylation and 6-his tag (baits) or pentamerizing COMP sequences followed by the beta-lactamase enzyme and a 6-his tag (preys) (31). The same process was followed for the *Plasmodium berghei* orthologs. All plasmids have been deposited in the Addgene plasmid repository (www.addgene.org/express).

### Production of the Recombinant Bait and Prey Proteins

All proteins were produced recombinantly as a monobiotinylated bait and a pentamerized prey by transient transfection of HEK293E (32) or HEK293-6E cells (33) essentially as described (30). All proteins were purified from the cell culture supernatant up to 6 days after transfection using Ni^2+^-NTA resin either using automated purification systems (GE Healthcare) or manually using Ni-NTA Agarose (Jena Biosciences) and dialyzed extensively against Hepes-buffered saline (HBS) or PBS.

### Normalization of Bait and Prey Proteins

The expression levels of biotinylated bait and β-lactamase-tagged prey proteins were quantified essentially as described (30). Briefly, for bait proteins, serial dilutions of the purified, dialyzed proteins were added to individual wells of a streptavidin-coated microtiter plate (NUNC). Proteins were captured and washed with HBS-T (HBS/0.1% Tween 20) followed by addition of 1 μg/ml mouse anti-rat Cd4 OX68 monoclonal antibody in HBS/0.2% bovine serum albumin (BSA) and washed with HBS-T. Proteins were detected by addition of a goat-anti-mouse alkaline-phosphatase conjugated antibody (Sigma A4656), washing followed by addition of 1 mg/ml alkaline phosphatase substrate 104 dissolved in diethanolamine buffer, and reading the absorbance at 405 nm in an automated plate reader (FluoStar Optima, BMG Labtech). For subsequent assays, the highest protein dilution that saturated the biotin binding capacity of the well was used. The activity of prey proteins was normalized by quantifying the activity of the β-lactamase enzyme in the prey protein tag. Serial dilutions were prepared in a 96-well flat bottom microtiter plate, and 20 μl was added to 60 μl of a 242 μM solution of the colorimetric β-lactamase substrate nitrocefin (Cayman Chemical Company). The kinetics of substrate hydrolysis were followed for 20 min and prey proteins normalized to an activity of ∼2 nmol min^−1^ for use in subsequent assays. To produce tetrameric preys to detect the CelTOS-PF3D7_0721100 interaction, a dilution series of monomeric biotinylated protein (similar to bait normalization) was incubated with 2 to 5 μg/ml alkaline phosphatase–coupled streptavidin (Sigma) for 45 min to allow the formation of tetramers and then used in an ELISA as described above. The lowest dilution that did not give a signal at 405 nm was used for AVEXIS.

### Western Blotting

Purified proteins were resolved by SDS-PAGE using Novex NuPAGE 4% to 12% Bis Tris precast gels (Life Technologies). Proteins were reduced by adding NuPAGE reducing agent to the sample and antioxidant (Life Technologies) to the gel running buffer according to the manufacturer's instructions. Proteins were blotted onto nitrocellulose membranes (Life Technologies) and protein-binding sites blocked using a 2% BSA solution. Biotinylated proteins were detected by incubating membranes with High-sensitivity Streptavidin–horseradish peroxidase (HRP) (PIERCE), washing, and addition of SuperSignal West Pico Chemiluminescent substrate (PIERCE) and developed on photographic film.

### Enzyme-Linked Immunosorbant Assay

ELISAs were performed by capturing biotinylated bait proteins into individual wells of streptavidin-coated 384-well plates (Nunc). Plates were washed for 30 min with 50 μl PBS-T (0.2% Tween) and blocked with PBS-2% BSA for a minimum of 3 h A volume of 20 μl of a bait protein diluted in PBS-2% BSA at a concentration previously determined as the amount required to saturate the biotin binding capacity of the well was added in triplicate and incubated for at least 16 h at 4 °C. Where required, a similar set of bait protein dilutions were heat inactivated for 30 min at 70 °C. Antisera from pooled Malawian adults (23) were centrifuged at 13,000 rpm for a minimum of 1 h, diluted in PBS-2% BSA and incubated with rocking for at least 16 h at 4 °C before adding to the antigen-coated plates for 1 h. Plates were washed 3× in PBS-T before addition of a 1:10,000 dilution of rabbit-anti-human polyvalent IgG/A/M (Sigma I8010) in PBS-2% BSA for 1 h, and washed in PBS-T, followed by 1:10,000 dilution of goat anti-rabbit Poly-HRP IgG/A/M (PIERCE) in PBS-2% BSA for 1 h. Plates were washed in PBS-T, and the HRP substrate ABTS (KPL) was added and absorption at 405 nm determined using an automated plate reader (FluoStar Optima, BMG Labtech).

### Avidity-Based Extracellular Protein Interaction Screening Assay

Systematic screening for protein interactions was performed essentially as described (22, 34). Briefly, normalized bait proteins were immobilized on streptavidin-coated 96-well microtiter plates (Nunc) and activity-normalized prey proteins added to the arrayed bait proteins and incubated at room temperature for 1 h. The plates were washed twice in HBS-T (0.2% Tween 20) and once in HBS before 60 μl of 125 μg/ml nitrocefin (Cayman Chemical Company) was added and the absorbance measured at 485 nm in a plate reader. When tetramerized preys were used, 1 mg/ml phosphatase substrate 104 in diethanolamine buffer was added and the absorbance measured at 405 nm. The rat Cd200–Cd200R interaction was used as a positive control, and a biotinylated Ox68 antibody that recognizes the Cd4-tag in the prey was used as a prey capture control. Some bait and prey proteins showed low levels of promiscuous binding in the screen (*e.g.*, bait 56 and prey 27) but because of their lack of specificity and weak binding signals were not investigated further.

### Experimental Design and Statistical Rationale

The goal of the protein interaction screening was to identify directly interacting proteins within the library of recombinant *P. falciparum* sporozoite membrane proteins. Sixty-four bait proteins were tested for interactions against 54 prey proteins; however, there were insufficient amounts of one of the baits (protein 53–SIAP-1) and therefore this bait was tested against 35 prey proteins. In total, 3437 binding tests were performed: 46 proteins were present in both prey and bait libraries and therefore tested in both bait–prey orientations; the other proteins were consequently tested in one orientation, making a total of 2379 unique interactions tested. The initial screen was performed once, but the identified interactions were repeated in triplicate and further characterized using surface plasmon resonance. Control baits and preys were present in all experiments; typically the rat Cd200–CD200R interaction is used as a positive control. Data are shown as mean absorbance readings with standard deviations to show reproducibility between replicates.

### Surface Plasmon Resonance

A Biacore 8K instrument (GE Healthcare) was used for the surface plasmon resonance studies essentially as described (35). In brief, biotinylated bait proteins were immobilized on a streptavidin-coated sensor chip (GE Healthcare). Approximately 400 RUs of the control bait (biotinylated rat Cd4d3+4) were immobilized in the flow cell used as a reference and the approximate molar equivalents of the query protein immobilized in the other flow cells. Purified analyte proteins were resolved by size exclusion chromatography on a Superdex 200 Increase 10/300 column (GE Healthcare) in HBS-P (10 mM Hepes, 150 mM NaCl, 0.05% v/v P20 surfactant) immediately before use in surface plasmon resonance (SPR) experiments thereby removing any aggregated protein that might influence kinetic measurements. The surface was regenerated with a pulse of 2 M NaCl at the end of each cycle, and all experiments were performed at 37 °C. Duplicate injections of the same concentration in each experiment were superimposable demonstrating no loss of activity after regenerating the surface. Binding data were analyzed in the manufacturer's Biacore 8K evaluation software version 1.1 (GE Healthcare).

### Cloning and Expression of *P. berghei* Orthologs

The *P. berghei* orthologs of the six identified interacting *P. falciparum* proteins were identified using PlasmoDB (36). Sequences were modified in the same way as described in Design of the Plasmodium Sporozoite Protein Library and the genes synthesized by GeneART. The synthetic plasmids were digested with NotI and AscI restriction enzymes (NEB) and were each subcloned into the bait and prey vectors. Recombinant proteins were produced by transient transfection of HEK293-E or 6E cells as described above.

### *P. berghei* Genetic Targeting

*P. berghei* targeting plasmids for *PIESP15* (*PBANKA_0209200*, PbGEM-268595) and *PBANKA_0800600*, PbGEM-331155 were obtained from the PlasmoGEM vector resource (plasmogem.sanger.ac.uk) (37). To make targeting plasmids that disrupt the *PBANKA_0522500* and *PBANKA_1241700* loci, oligonucleotides were designed to amplify 5′ and 3′ homology regions of each gene. Each homology arm was approximately 1 kbp long and spans the 5′ UTR into the open reading frame, and from inside the open reading frame into the 3′UTR of each gene, respectively. Restriction sites were added to the primer sequence to enable cloning of the PCR products. PCRs were performed using Kapa HiFi HotStart Ready Mix (Kapa Biosciences), and genomic DNA isolated from *P. berghei* GFP-LUC parasites (38) was used as a template. PCR conditions were 5 min at 95 °C followed by 35 cycles of 95 °C for 30 s, 55 °C for 30 s and 68 °C for 1 to 3.5 min, and a final extension of 10 min at 68 °C. PCR products were sequentially cloned into the KpnI and XmaI or the XhoI and SacII restriction sites of the pR6K attL1-3xHA-h*DHFR*-*yfcu*-attL2 plasmid backbone (39) and used to transfect *P. berghei* parasites.

### Transfection and Cloning by Limiting Dilution

Recombinant parasites were generated by transfecting a parental “wildtype” transgenic *P. berghei* strain expressing green fluorescent protein (GFP) and luciferase (38). Transfections were done essentially as described (40). Briefly, 6- to 12-week-old Balb/C or Theiler's original mice were used for routine propagation of the parasites. Prior to transfection, 10 μl DNA was digested with NotI restriction enzyme (NEB) overnight at 37 °C to release the transfection cassette from the plasmid backbone. DNA was purified by ethanol precipitation and resuspended in 10 μl dH_2_O. Parasites were cultured *in vitro* for 20 to 22 h to obtain synchronized schizonts. Schizonts were purified using a 55% Nycodenz (Sigma)/PBS gradient and washed once in RPMI1640 medium. Pelleted schizonts were resuspended in P3 Primary cell Nucleofector (Lonza, 18 μl/transfection), and 25 μl of this mix was added to 5 μl of digested DNA. Twenty microliters of the mix was then added to an individual well of a 16-well Nucleocuvette Strip and pulsed using the AMAXA 4D-Nucleofector electroporator, program FI-115. Parasites were immediately resuspended in 100 μl incomplete RPMI1640 medium and injected into the tail vein of Balb/c mice. On the following day in the morning, pyrimethamine (0.07 mg/ml, pH 4.5) was added to the drinking water for parasite selection. Blood smears were taken from day 7 after transfection to monitor parasitemia, and when parasitemia reached 0.5% to 1%, blood was collected for stocks and genotyping. Subsequently, a drop of blood was taken from a mouse infected with the transfected parasites and parasites were cloned by limiting dilution. Clonality (absence of the WT locus) was confirmed by genotyping, and cloned parasites were then used for experiments.

### Genotyping of *P. berghei* Parasites for Validation of Gene Disruption

To confirm integration of the transfection cassette into the genome of the parasite, we designed specific oligonucleotides for each target gene, annealing to the chromosome just outside the homology arm of the plasmid (primer GT). This was paired with a universal primer that anneals to the *yfcu* cassette in the targeting plasmid (primer GW2a or b). To detect absence or presence of the WT locus, a gene-specific primer located inside the gene sequence of the target gene (primer QCR1) was paired with a reverse primer in the homology arm (QCR2). Primers GW2 and QCR2 were then combined to detect the transfection plasmid, which could be either integrated or not integrated in the genome. Because the product size for the primer combination *PbPIESP15*-GW2+GT was too large, two PCR reactions were done to amplify one half of the fragment each. Therefore, primer GW2b was combined with primer PIESP15-rev2 and primer GT was combined with primer *PbPIESP15*-fwd2. Sequences for primers QCR1, QCR2, and GT for *PbANKA_0800600* and *PbPIESP15* were obtained from the PlasmoGEM web page. All primer sequences are listed in Table S2. PCR reactions were performed using Kapa HiFi HotStart Ready Mix (Kapa Biosciences), and cycling conditions were 5 min at 95 °C followed by 35 cycles of 95 °C for 30 s, 55 °C for 30 s, and 68 °C for 1 to 3.5 min and a final extension of 10 min at 68 °C. Samples were separated on a 0.8% Agarose gel using Hyperladder 1kb (Bioline) as a size standard.

### *In Vivo* Phenotyping of Transgenic *P. berghei* Parasites

*Anopheles stephensi* mosquitoes were allowed to feed on anaesthetized *P. berghei-*infected Balb/C mice for no more than 15 min and placed in an incubator at 19 °C thereafter. Nine to 11 days after infection, mosquitoes were anesthetized and kept on ice and midguts were dissected in PBS. Only mosquitoes with developed eggs were dissected to ensure mosquitoes had taken a blood meal at the time of feeding. Images were taken by fluorescence microscopy and used to quantify GFP-positive oocysts. Oocysts were counted manually using the cell counter plugin in ImageJ 1.45s software. To quantify the number of sporozoites in salivary glands, mosquitoes were anesthetized 21 days after infection and salivary glands dissected in PBS. Salivary gland sporozoites from 15 to 20 mosquitoes were released by homogenizing the glands with a micropestle, and salivary gland debris were pelleted by centrifugation for 5 min at 100*g*. Sporozoites were subsequently counted in a hemocytometer. Groups of four mice were infected by intravenous injection of 5000 salivary gland sporozoites per mouse and the development of the parasites was followed in the whole animal every 24 h for 72 to 96 h by an *in* *vivo* imaging system (IVIS) imaging. For imaging, mice were injected with 200 μl D-luciferin (30 mg/ml in Dulbecco's phosphate buffered saline, Source Biosciences) and imaged under isoflurane anesthesia 10 min later using an IVIS Spectrum Imaging System (Perkin Elmer).

Once parasites were observed in the blood by IVIS (usually 72 h post sporozoite injection), thin blood smears were taken daily for a minimum of 5 days and stained using Giemsa to monitor blood-stage parasitemia. To determine parasitemia, at least 500 red blood cells were counted and parasitemia was calculated as a percentage of infected red blood cells per total number of red blood cells; percentages were then used for growth curves.

### Production of Polyclonal Antisera

For the production of polyclonal antisera, proteins were expressed as nonbiotinylated monomers by transient transfection of HEK293 cells, purified using the C-terminal his tag, and dialyzed against PBS. For immunization, 50 μg protein per animal was adjuvanted in Alhydrogel (Invivogen) and delivered intraperitoneally into 6- to 8-week-old female Balb/C mice. Two subsequent boosts were given with 20 μg per animal at 2 and 4 weeks after the initial injection. Sera were obtained 1 week after the final immunization boost by cardiac bleed and centrifugation of the whole blood after clotting.

### *P. falciparum* Cell Invasion Assays

HC-04 cells were routinely cultured in Dulbecco's Modified Eagle Medium: Nutrient Mixture F-12 (DMEM-F12; ThermoFisher Cat#31330038), supplemented with 10% heat-inactivated fetal bovine serum and 1× Penicillin/Streptomycin. These cells were incubated at 37 °C under 5% CO_2_ and split using trypsin when 80% confluency was reached. Twelve hours before the assay, 50,000 cells per well were seeded in a collagen-coated 96-well flat-bottom plate (Corning Cat# CLS3603-48EA). On the day of the experiment, salivary glands of *P. falciparum* NF-135-infected mosquitoes were dissected in 1 ml DMEM-F12 media and crushed using a glass pestle to liberate the sporozoites. Sporozoite numbers were determined using a hemocytometer. Media from each of the hepatocyte-seeded wells were removed and replaced with media containing 50,000 sporozoites per well. The plate was centrifuged at 3000 rpm for 10 min and incubated at 37 °C under 5% CO_2_ for 3 h. After 3 h, the supernatant was removed and each well rinsed three times with PBS and trypsinized for 5 min to obtain single-cell suspension. The trypsinized sample was neutralized in equal volumes of PBS supplemented with 10% FCS and transferred to a 96-well V-bottom plate (Costar Cat#3897). Samples were spun down at 1700 rpm for 4 min. Supernatants were removed, and pellets were resuspended in fixation reagent as per manufacturer's instructions (eBioscience Foxp3/Transcription factor staining buffer set; ThermoFisher Cat# 00-5523-00) and incubated at 4 °C for 30 min. Permeabilization buffer was added to each well after the incubation, and the samples were pelleted at 1700 rpm for 4 min. The supernatant was removed, the pellet was resuspended in permeabilization buffer containing 3SP2 (antibody against PfCSP 1:400 dilution) conjugated to FITC and incubated at 4 °C for 30 min. Permeabilization buffer was added to each well after the incubation, and the samples were pelleted at 1700 rpm for 4 min. The supernatant was removed, and the pellets were resuspended in 1% paraformaldehyde in PBS. Each sample was read by fluorescent activated cytometry sorting to determine the percentage of FITC-positive (infected) cells.

### Immunostaining of *P. berghei* Sporozoites

To determine the amount and subcellular localization of CSP, salivary gland sporozoites were dissected from infected *A. stephensi* mosquitoes 21 to 30 days after blood feeding on *P. berghei*-infected mice. Sporozoites were isolated by homogenizing the glands with a micropestle and centrifugation for 5 min at 100*g* to pellet salivary gland debris. Sporozoites were subsequently counted in a hemocytometer, and 10,000 sporozoites each were placed onto round 13-mm coverslips and air dried. Coverslips were then stored at −80 °C with dessicant until used for immunostaining. For immunostaining, the coverslips were left to reach room temperature before being removed from the bag containing desiccant. Coverslips were then placed into individual wells of a 24-well plate (Nunc), and parasites were fixed with a 4% formalin solution in PBS for 10 min on ice. After washing twice in PBS, the coverslips were incubated with 0.1% Triton in PBS on ice for 15 min to permeabilize the parasites. All slides were washed twice in PBS, 1% BSA (Sigma) and incubated with anti-CSP monoclonal antibody, 1:1000 in PBS, 1% BSA for 1 h at room temperature. After washing three times in PBS, 1% BSA parasites were incubated with Cy3 goat anti-mouse IgG (Life Technologies A10521), 1:500 in PBS, 1% BSA for 30 min at room temperature, protected from light. Coverslips were subsequently washed in PBS, 1% BSA and mounted on microscope slides with Vectashield HardSet containing DAPI (Vectorlabs).

## Results

### A Library of *P. falciparum* Sporozoite Surface and Secreted Proteins

**Fig. 1:**
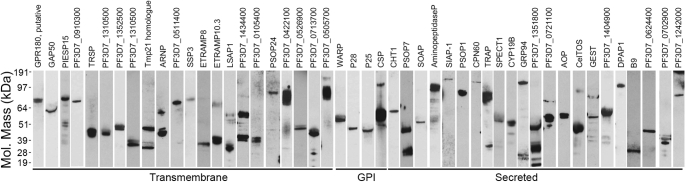
**A library of recombinant *P. falciparum* sporozoite cell surface and secreted proteins.** The library of sporozoite receptor ectodomains was expressed as soluble recombinant enzymatically biotinylated proteins in HEK293 cells, and the purified proteins were resolved by SDS-PAGE under reducing conditions. Proteins were blotted and detected using streptavidin–horseradish peroxidase.

To create a recombinant protein library representing the cell surface receptor repertoire and secretome of the *P. falciparum* sporozoite, we analyzed published proteome and transcriptome datasets from both *P. falciparum* and *Plasmodium yoelii* sporozoites (25, 26, 41). To identify proteins that are likely to be displayed on the surface of the sporozoite or secreted, we selected those that contain a predicted N-terminal signal peptide and/or transmembrane domain using a set of bioinformatic tools. The resulting list of 102 proteins included 57 proteins that contained one or more transmembrane-spanning regions, 11 that encoded a C-terminal hydrophobic sequence for the addition of a GPI lipid anchor, and 34 that had no obvious way of being tethered to a membrane, and so were predicted to be secreted (Table S1). Of these 102 proteins, 25 are also known to be expressed on the blood stages (such as P38, RhopH3, and MSP1) and already represented within our library of previously published merozoite proteins (23, 24). For the remaining 77 proteins, we designed expression constructs in a similar manner by truncating the proteins just prior to the predicted transmembrane region or GPI-anchor and using gene synthesis to codon optimize the coding regions for expression in human cells (23). Of these, 69 genes could be successfully synthesized and subcloned into the mammalian expression plasmid (Table S1) and were subsequently expressed as Cd4d3+4-tagged enzymatically monobiotinylated bait proteins by transient transfection of suspension-grown HEK293 cells. Using this approach, 58 of the 69 proteins (84.1%) could be detected after his-tag purification from the spent cell culture medium by ELISA. As is typical for this approach, for those proteins that were detectably expressed, levels varied widely across several orders of magnitude but could be as high as ∼50 μg/ml for proteins such as TRAP and CSP. Forty-eight proteins could be produced in sufficient quantities to be used in the subsequent experiments and were resolved by SDS-PAGE and detected by anti-biotin Western blotting to determine their integrity (Fig. 1). With the exception of three proteins, PSOP7, B9, and PF3D7_0624400, whose major species was smaller than expected, the proteins were expressed at the expected mass, although some proteins did show evidence of being processed. Those proteins that could not be detected or were expressed at very low levels included proteins that localize to the rhoptries (RALP1, ARNP, and MAEBL), both the LCCL-domain containing secreted proteins LAP2 and LAP4, two members of the TRAP family (CTRP and TREP), and two members of the 6-cys family that are also expressed on gametes (P47 and P48/45). We have previously observed that proteins localized to the rhoptries of merozoites are also poorly expressed in this system suggesting that a rhoptry-specific chaperone may be required for expression using this approach (23).

### The Recombinant Sporozoite Proteins Are Immunoreactive and Contain Conformational Epitopes

**Fig. 2:**
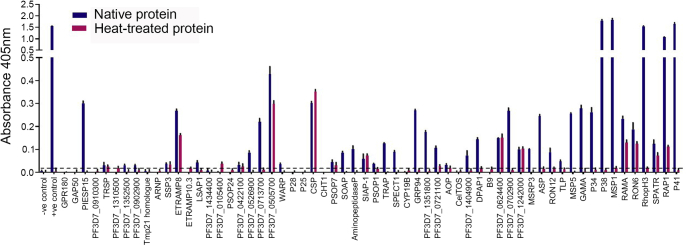
**The recombinant *P. falciparum* sporozoite proteins contain heat-labile conformational epitopes.** The immunoreactivity of the recombinant sporozoite proteins to immune serum was quantified by ELISA. Biotinylated proteins were normalized and captured on a streptavidin-coated microtiter plate and probed with pooled immune sera from Malawian adults (*blue*). Reduced response of immune serum to heat-treated (70 °C for 30 min) proteins (*pink*) demonstrates the presence of heat-labile conformational epitopes. Note that the 11 proteins between MSP5 and P41 that are grouped on the right of the graph are additionally expressed in blood-stage parasites. Bar charts show means ± SEM; *n* = 3. Positive immunoreactivity was defined as mean responses >3 SEM above the averaged control (*dashed line*).

To systematically evaluate which of the recombinant proteins within the sporozoite library contained conformational epitopes, we quantified the immunoreactivity to purified immunoglobulins pooled from adult donors living in a *P. falciparum* malaria-endemic region. Over half (35/60, 58%) of the proteins were recognized by the pooled immune serum (Fig. 2). We found that the overall immunoreactivity to sporozoite antigens was lower than for merozoite antigens (23), as expected given that the immune system is exposed to many orders of magnitude fewer sporozoites than merozoites during an infection. The most immunoreactive proteins were those whose expression is not restricted to the sporozoite stage but that are also expressed during the blood stages. Sporozoite proteins that are not expressed by the blood stages generally exhibited a lower immunoreactivity with CSP, PIESP15, ETRAMP8, and three conserved proteins with unknown function: PF3D7_0702900, PF3D7_0713700, and PF3D7_0505700 being the most immunodominant. Except for CSP, the immunoreactivity of the immune serum to heat-treated proteins was either completely lost or reduced compared with the untreated proteins, demonstrating that the immune serum recognized conformational epitopes and suggesting that most proteins were folded (Fig. 2). The lack of heat-labile epitopes in recombinant CSP is consistent with the known immunodominance of the repetitive “NANP” repeats, which are predicted to be natively unstructured (42). These data demonstrate that the majority of soluble recombinant *P. falciparum* sporozoite proteins expressed in mammalian cells contain conformational epitopes and represent a valuable resource for studying the molecular pathology of *P. falciparum* infections.

### Identification of *P. falciparum* Sporozoite Surface Protein Complexes by Systematic Protein Interaction Screening

**Fig. 3:**
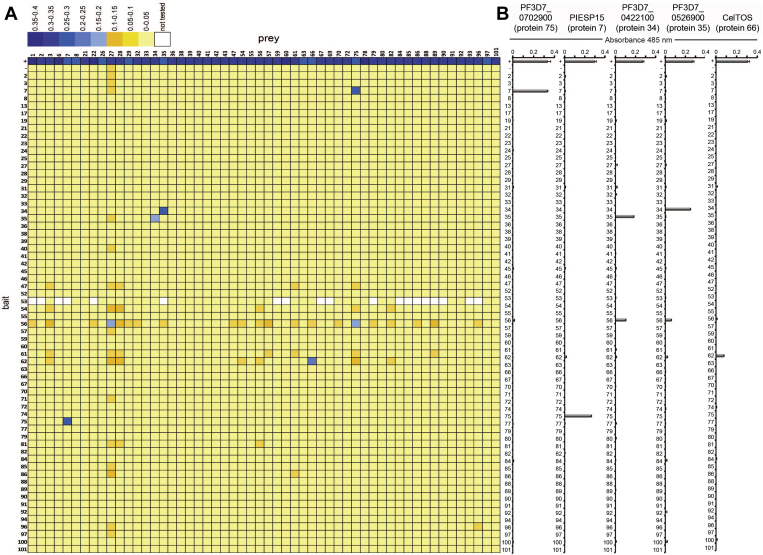
**Identification of three *P. falciparum* sporozoite complexes by systematic extracellular protein interaction screening.***A*, the library of cell surface and secreted sporozoite proteins was expressed as biotinylated bait and enzyme-tagged pentameric prey proteins and systematically screened for interactions using the AVEXIS assay. The results are presented as a quantitative binding grid. The biotinylated baits (arranged vertically according to the numbers in Table S1) were immobilized in individual wells of streptavidin-coated microtiter plates and tested for direct binary interactions with the same proteins expressed as pentameric β-lactamase-tagged preys (arranged horizontally). Binding by specific baits is quantified by detecting the capture of prey proteins and hydrolysis of the colorimetric β-lactamase substrate nitrocefin. *B*, the five named prey proteins that interacted with a bait were retested in triplicate against the arrayed baits and the interactions confirmed. Controls were: positive (+) Cd200 bait -Cd200R prey; negative (−) Cd200 bait with corresponding sporozoite protein prey. Bars represent mean ± SD; *n* = 3.

To identify *P. falciparum* sporozoite extracellular protein complexes we used the AVEXIS assay, which detects direct interactions between soluble recombinant ectodomains expressed as biotinylated bait and highly avid enzyme-tagged prey proteins (22). To produce the sporozoite library as prey proteins, the ectodomains were subcloned into a plasmid containing tags for pentamer formation and expressed in HEK293 cells. As expected, the levels of activity varied and 54 prey proteins were expressed at sufficient levels after their activities had been normalized to the threshold level required for the AVEXIS assay (Table S1). In total, 64 bait proteins were systematically tested for interactions against the 54 prey proteins (Fig. 3*A*). One of the baits (SIAP-1 - protein 53) was expressed at low levels and therefore tested against just 35 prey proteins so that a total of 3437 binding tests were performed. Forty-six of the proteins were present in both bait and prey libraries and consequently tested in both bait–prey orientations; the remaining proteins were therefore tested in a single orientation only, resulting in a total of 2379 unique interactions tested. We identified three interactions: the first between a type I cell surface protein called PIESP15 and a secreted protein: PF3D7_0702900, a second between two members of the p24 family of proteins (PF3D7_0422100 and PF3D7_0526900), and the third between CelTOS and a predicted secreted protein (PF3D7_0721100). The PIESP15-PF3D7_0702900 and p24 family interactions were identified in both bait–prey orientations in the initial screen; however, because the PF3D7_0721100 protein did not express at sufficient levels in the prey format, the CelTOS-PF3D7_0721100 interaction was initially identified in one orientation. For those five prey proteins that interacted with a specific bait, we repeated the binding assays against all the baits in triplicate and confirmed the interactions (Fig. 3*B*). The interaction between CelTOS and PF3D7_0721100 was confirmed using a modified assay where monomeric biotinylated proteins were clustered around an alkaline phosphatase–streptavidin conjugate to form avid tetrameric preys, which then could be used to probe immobilized baits to test for interactions (43). Using this method, we could show binding of CelTOS and PF3D7_0721100 in both orientations relative to controls (Fig. S1).

### The Interactions Are Conserved Between Orthologous Proteins in *P. berghei*

**Fig. 4:**
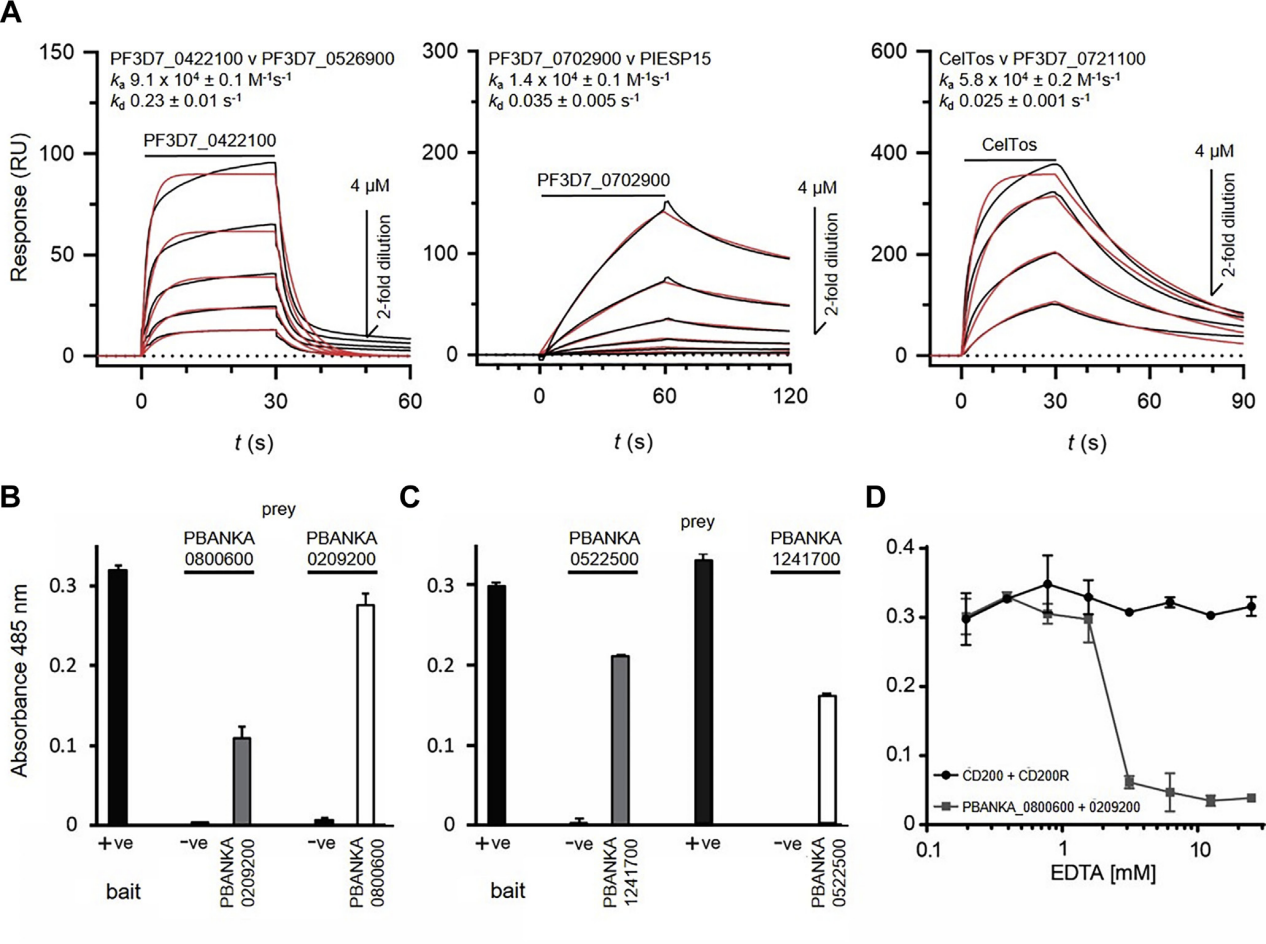
**Biophysical characterization of the three sporozoite complex interactions and conservation of interactions with orthologous proteins in *P. berghei***. *A*, biophysical characterization of the three *P. falciparum* sporozoite interactions by surface plasmon resonance. For each interacting pair, one protein was immobilized on the sensor chip and twofold serial dilutions of the indicated corresponding binding partner was injected to quantify binding parameters. The raw sensorgram data (*black lines*) were fitted to a 1:1 binding model (*red lines*) to derive the association (*k*_a_) and dissociation (*k*_d_) rate constants. *B*, the entire ectodomains of the *P. berghei* orthologs of PIESP15 (PBANKA_0209200) and PF3D7_0702900 (PBANKA_0800600), as well as PF3D7_0422100 (PBANKA_0522500) and PF3D7_0526900 (PBANKA_1241700) (*C*), were expressed as both bait and prey proteins and shown to directly interact using the AVEXIS assay in both bait–prey orientations. The rat Cd200–Cd200R interaction was used as a positive control (+ve); negative control bait (−ve) was Cd200. (D) The PIESP15–PBANKA_0800600 interaction but not the control Cd200–Cd200R interaction was inhibited in a dose-dependent manner by addition of EDTA as detected by AVEXIS. Bars and data points indicate means ± SD; *n* = 3.

To further characterize these interactions, we first used SPR to establish whether the proteins could directly interact and quantify their biophysical binding parameters. For each interacting pair, one protein was purified and serial dilutions injected over its binding partner expressed as a biotinylated protein and immobilized on a streptavidin-coated sensor chip. Clear binding was observed for each interaction, and the association and dissociation binding parameters were quantified by fitting the series of binding traces to a 1:1 binding model (Fig. 4*A*). As expected, the two interactions involving a protein predicted to be secreted exhibited slower dissociations than the membrane-tethered p24 complex proteins (21). Genome analysis revealed that the six proteins forming complexes had identifiable orthologs in the genomes of all *Plasmodium* species sequenced to date (36). To determine if the protein interactions were conserved across species, we expressed the orthologs of these proteins from the rodent parasite *P. berghei* as both baits and preys and tested if they could directly interact using the AVEXIS assay. We could detect robust interactions between *Pb*PIESP15 and *Pb*ANKA_0800600 and the two *P. berghei* p24 complex proteins in both bait–prey orientations (Fig. 4, *B* and *C*). We were unable to determine if the interaction between CelTOS and PF3D7_0721100 was conserved between the *P. berghei* orthologs of these proteins because repeated attempts to produce the *P. berghei* ortholog of PF3D7_0721100 failed; therefore, this interaction was not investigated further. These data demonstrate evolutionary conservation of the interaction interfaces, which is striking since the amino acid sequence identity of the extracellular regions was relatively modest: 58% and 64% for the two p24 complex proteins and 42% and 75% for PIESP15 and PF3D7_0702900, respectively. We next sought to characterize the biochemical interaction between PIESP15 and PF3D7_0702900 in more detail. Both proteins contain recognizable protein domains in their extracellular regions: PIESP15 has an L-type lectin domain, so named because it has homologies to lectins found in leguminous plants (44), and PF3D7_0702900 contains tandem EF-hand domains. It seemed unlikely that the PIESP15–PF3D7_0702900 interaction involved carbohydrate binding because *Plasmodium* parasites lack the glycosyltransferase enzymes necessary for complex carbohydrate modifications (45, 46), and because of the design of the recombinant proteins the only predicted glycosylation was present within the Cd4 protein tag common to all bait proteins. Both L-type lectin and EF hand domains are known to require calcium ion cofactors in their binding sites (47, 48), and consistent with this we observed that the PIESP15- PF3D7_0702900 interaction was inhibited in a dose-dependent manner in the presence of the divalent ion chelator, EDTA (Fig. 4*D*).

### Both Genes Encoding p24 Complex Proteins Are Required for *P. berghei* Liver-Stage Infection

**Fig. 5:**
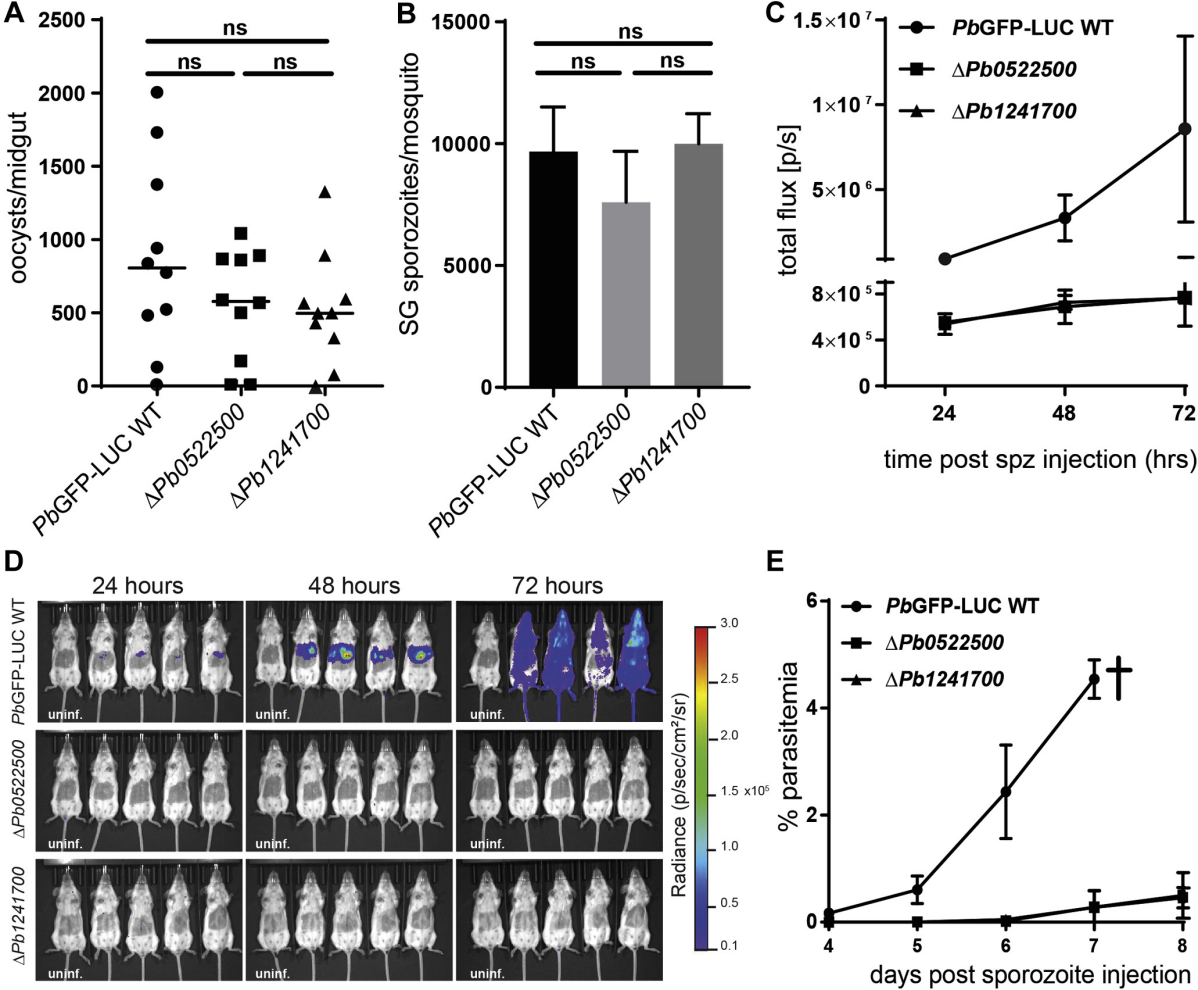
**Both genes encoding the interacting p24 complex proteins are required for *P. berghei* mammalian host liver-stage infection.***A*, the number of oocysts per midgut in *A. stephensi* mosquitoes infected with *P. berghei* Δ*PBANKA_1241700* or Δ*PBANKA_0522500* were counted and found to be not significantly different from the GFP-luciferase transgenic wildtype parental strain. Data points are individual midguts; median is indicated and statistical comparisons were performed using the Mann–Whitney test; *p*-values > 0.05 were considered nonsignificant (ns). *B*, average number of salivary gland (SG) sporozoites per mosquito. About 15 to 20 mosquitoes were dissected, and sporozoites isolated from the salivary glands were counted. The columns represent the average number of salivary gland sporozoites across four independent experiments. Columns represent the mean with SD; statistical analysis was performed using an unpaired *t* test. *C*, *P. berghei* Δ*PBANKA_1241700* or Δ*PBANKA_0522500* parasites do not establish robust liver-stage infections in mice compared with wildtype control. Groups of four mice were infected by intravenous administration of 5000 isolated salivary gland sporozoites, and the resulting parasitemia was quantified in the whole animal using luciferase-based bioluminescence using an *in vivo* imaging system. Data points are mean ± SD; *n* = 4. *D*, normalized bioluminescence images collected on each day after infection; the left-most animal in each cohort is an uninfected control. *E*, the prepatency period before detection of blood-stage parasitemia is significantly delayed in Δ*PBANKA_1241700* and Δ*PBANKA_0522500* parasites relative to the wildtype control. WT animals were culled on day 7 because they showed signs of disease (indicated with a *cross*). Asexual blood-stage parasitemia was quantified by microscopic analysis of blood smears taken between 4 and 8 days after infection. Shown are the results from one of two independent experiments with similar results. Data points are mean ± SD; *n* = 4. The prepatency period was less than 4 days for the parental line and 7.0 ± 0.7 (mean ± SD; *n* = 4) for Δ*PBANKA_0522500* and 7 ± 1 for Δ*PBANKA_1241700.*

The cross-species conservation of both the PIESP15-PF3D7_0702900 and p24 complex protein interactions suggested that *P. berghei* was a suitable and experimentally tractable model and enabled us to further validate these interactions by asking if parasites lacking genes encoding interacting proteins had similar infection phenotypes. To make gene-deficient parasites for all four genes, we used existing plasmids from the PlasmoGEM resource to target both *PbANKA_0800600* and *PIESP15* (49). Gene targeting plasmids for the *P. berghei* orthologs of both p24 complex genes, *PBANKA_1241700* and *PBANKA_0522500*, were not available, so these were designed and constructed (Fig. S2). Gene-deficient parasites for all four genes were made by electroporating erythrocytes infected with a GFP-luciferase-transgenic “wildtype” *P. berghei* parental line and then cloned by limiting dilution. Parasites were genotyped to confirm recombination at the targeted locus (Figs. S2, B and C and S3). Mosquitoes were infected with the cloned parasite lines, and the infection was quantified throughout the life cycle in both the mosquito vector and mammalian host. We observed that both the Δ*PbPIESP15* and Δ*PbANKA_0800600* parasite lines had no overt infection phenotype in either the mosquito or in mice compared with the wild-type parental line (Fig. S4). This lack of infection phenotype was consistent with the inability of polyclonal antibodies to both PIESP15 and PF3D7_0702900 to prevent invasion of *P. falciparum* sporozoites in human cells (Fig. S5). By contrast, *P. berghei* parasites lacking either *PBANKA_1241700* or *PBANKA_0522500* infected mosquitoes indistinguishably from the wildtype line (Fig. 5, *A* and *B*), whereas intravenous infections of mice with salivary gland sporozoites resulted in a striking reduction in the number of parasites in the liver stage of infection for both genes (Fig. 5, *C* and *D*). This was confirmed by a significant delay in the appearance of parasites in the blood of infected mice (Fig. 5*E*).

## Discussion

In this study we have compiled a library of soluble recombinant forms of the membrane-embedded and secreted proteins from the *P. falciparum* sporozoite. We have shown how this resource can be used to characterize the host humoral immune response in patients with malaria and identify protein complexes that are important for sporozoite function. A key feature of this resource is that we have produced the proteins in a mammalian expression system to increase the chances that the proteins contain structurally critical posttranslational modifications such as disulfide bonds and thereby adopt their native conformation, which is essential for retaining extracellular binding functions. A similar library representing the secreted and membrane-embedded proteins from the *P. falciparum* merozoite has proven very valuable to define the serology of the infected host (50), identify both parasite–parasite (9, 13) and host–parasite protein interactions (51, 52, 53, 54), as well as blood-stage vaccine candidate screening (55). An important facet of this protein library is that many antigens can be assessed in parallel so that they can be directly compared in a systematic manner rather than the more common testing of proteins individually, which introduces technical variation that makes comparison very difficult. We therefore envision that this resource will be an important tool for the malaria community and have an impact in defining a better molecular understanding of the basic infection biology of the *P. falciparum* sporozoite; ultimately, these findings will contribute toward providing new opportunities for therapeutic interventions.

Of the three interactions we describe, the interaction between the p24 complex proteins PF3D7_0526900 and PF3D7_0422100 was of particular interest because *P. berghei* parasites lacking the orthologs of either of these genes exhibited strong sporozoite *in vivo* infection phenotypes. p24 proteins contain a GOLD (GOLgi Dynamics) domain, and four can be readily identified from their primary protein sequence in *Plasmodium* spp. genomes. Most p24 family proteins are ubiquitously expressed, although some may show elevated expression in different developmental contexts, and at least three are abundant in sporozoites and therefore represented in our library. We only observed interactions between two of these three proteins suggesting that there is binding specificity within the family, which is most likely mediated by the regions of predicted coiled coil in the lumenal domain (56). p24 family proteins are mainly localized to the membranes of the early secretory pathway, and experiments in yeast have suggested that they play major roles as cargo receptors for GPI-linked proteins. Their lumenal (ectodomain) regions function as a lectin by binding the oligo-mannose glycans in the GPI anchor (57) while their short cytoplasmic tails nucleate the formation of both COPI and COPII vesicle coats through their dilysine and phenylalanine-containing motifs, respectively (58). One likely molecular interpretation for the phenotypes of sporozoites lacking the interacting p24 family proteins is that they are unable to appropriately traffic GPI-anchored proteins that are important for sporozoite invasion of host cells to the sporozoite surface. One obvious candidate is CSP, but we did not observe any obvious difference in the localization or amount of CSP at the surface of sporozoites that lacked either *PBANKA_1241700* or *PBANKA_0522500* (Fig. S6). It is known that p24 family proteins are not essential for GPI-anchored protein trafficking since an engineered yeast strain lacking all known p24 family proteins only has a subtle phenotype that affects the rate and fidelity of protein trafficking (59). This suggests that most GPI-anchored proteins in yeast necessary for laboratory growth can be trafficked by “bulk flow” through the secretory pathway. Perhaps because CSP is so abundant at the sporozoite surface, it mainly relies on bulk flow for secretion and so is not affected by the loss of p24 family proteins. The infection phenotype we observed may therefore be due to mistrafficking of one or more of the several other GPI-anchored proteins that are expressed by sporozoites.

Of the two other interactions that we identified, we were able to show that *P. berghei* parasites genetically targeted for genes encoding the interacting proteins *PIESP15* and *PbANKA_800600* had no overt infection phenotypes, although because sporozoites were delivered by intravenous injection, there remains the formal possibility of a phenotype in sporozoite behavior in the dermis. These findings are consistent with the lack of *in vivo* blood-stage growth phenotypes for knockouts of these genes in *P. berghei* as part of a large-scale genetic screen (49), as well as a previous smaller-scale genetic screen focused on identifying genes encoding cell surface and secreted proteins required by the ookinete to infect mosquitoes (60). There is proteomic evidence from several studies that these proteins are not restricted to sporozoites in *P. falciparum* but can be detected in other stages including gametocytes (61) and blood stages (62). Of interest, insertional mutagenesis genetic screens have shown that both *PIESP15* and *PF3D7_0702900* are very important for *in vitro* blood-stage culture in *P. falciparum* providing evidence that these genes may play important roles in *P. falciparum* in other lifecycle stages (63). It is worth noting that the genes encoding PF3D7_0702900/*Pb*ANKA_0800600 are currently annotated as a “putative centrin” in gene databases, which appears to be a computational prediction based on very low (<30%) protein sequence identity. There is no functional evidence to support this claim and both PF3D7_0702900/*Pb*ANKA_0800600 have confident signal peptide predictions strongly suggesting an extracellular localization, which would not be compatible with the required cytoplasmic localizations of centrin and its known role in centrosome biology. We were not able to further investigate the functional role of the interaction between CelTOS, a protein of considerable interest owing to its role in mosquito infection and cell traversal (64), and PF3D7_0721100 in *P. berghei* because initial attempts to genetically target the *P. berghei* ortholog *PBANKA_0618600* did not result in any recombinants and we were unable to express the protein in a recombinant form.

In summary, we have produced a library of secreted and membrane-embedded proteins that are expressed by the *P. falciparum* sporozoite using a mammalian expression system. We believe that these proteins will be particularly useful to the malaria research community because of the challenges in obtaining large amounts of sporozoite material and solubilizing membrane-embedded proteins in their native conformation. Given that targeting the sporozoite stage either as attenuated whole sporozoites or as a sporozoite surface protein-based subunit vaccine has shown promise in malaria vaccine development (6), gaining a greater understanding of these proteins and how they function will not only advance a molecular understanding of sporozoite infection biology but is also likely to provide important information to improve sporozoite-based malaria vaccine design.

## Data availability

All the data are available within the article and associated supplementary information. Expression plasmids encoding the proteins have been deposited and are available from the Addgene repository (www.addgene.org).

## Conflict of interest

The authors declare no competing interests.
