# Supplementary material for: Systematic Identification of Plasmodium Falciparum Sporozoite Membrane Protein Interactions Reveals an Essential Role for the p24 Complex in Host Infection
Source: Mol Cell Proteomics. 2021 Jan 27;20:100038. doi: 10.1074/mcp.RA120.002432 (PMC7950211; doi:10.1074/mcp.RA120.002432)
Supplement: Figures S1 to S6 and Tables S1 & S2 [file mmc1.pdf]

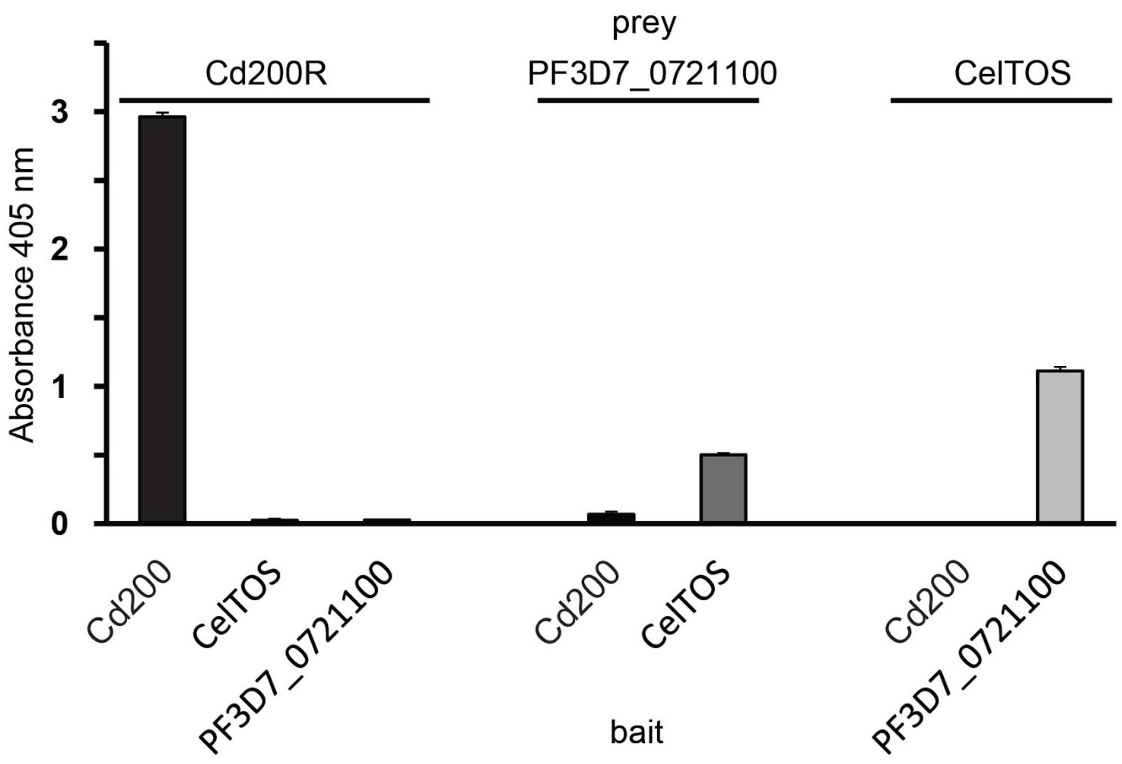

Figure S1. Confirmation of the interaction between *P. falciparum* CelTOS and PF3D7_0721100 using a modified avidity-based interaction assay. CelTOS and PF3D7_0721100 were shown to directly interact in both bait-prey orientations using a modified avidity-based interaction assay. The entire ectodomains of *P. falciparum* CelTOS and PF3D7_0721100 were expressed as monomeric biotinylated bait proteins and captured on a streptavidin-coated microtitre plate. Highly avid tetrameric prey binding probes were made by complexing the biotinylated monomers to a streptavidin alkaline phosphatase conjugate and adding normalised amounts to bait-coated wells. Interactions were quantified by adding phosphatase substrate and measuring the absorbance at 405 nm. The rat Cd200-Cd200R interaction was used as a positive control (+); negative control bait (-) was Cd200. Bars indicate means ± SD; $n = 3$. Shown are the results from one of two independent experiments with similar results.

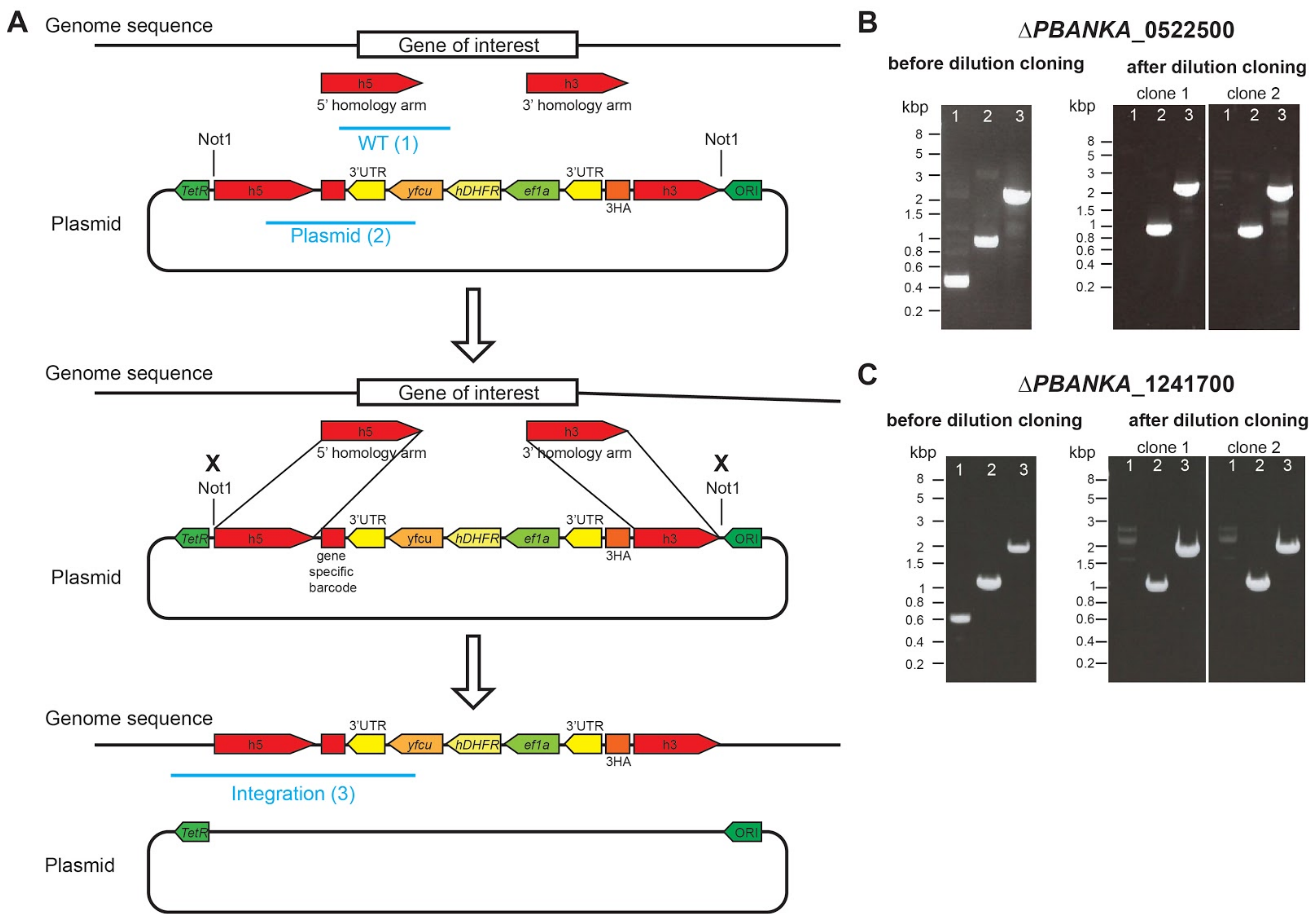

Figure S2. Overview of cloning strategy for Δ*PBANKA_0522500* and Δ*PBANKA_1241700* vectors and genotyping of parasites. (**A**) Generalised schematic overview of the cloning strategy used to generate plasmids used to target *PBANKA_0522500* and *PBANKA_1241700* genes in *P. berghei* parasites. 5' and 3' homology arms were amplified from genomic DNA and cloned into the pR6K attL1-3xHA-h*DHFR*-*yfcu*-attL2 plasmid backbone and transfected into *P. berghei* after digestion with the NotI restriction enzyme (X) to release the gene targeting cassette. The cassette is integrated into the genome by homologous recombination and replaces the gene of interest. The fragments generated by PCR to confirm deletion of the WT locus and integration of the plasmid into the genome are indicated in blue. (**B, C**) Genomic DNA was isolated from parasites before (left) and after (right) dilution cloning of Δ*PBANKA_0522500* (**B**), and Δ*PBANKA_1241700* (**C**) parasites. Polymerase chain reactions were performed with primers designed to detect the WT locus (1), the transfection vector (2) and the recombined locus (3). After dilution cloning, the WT genes were not detected demonstrating that the parasite lines are clonal and only contain recombinant parasites.

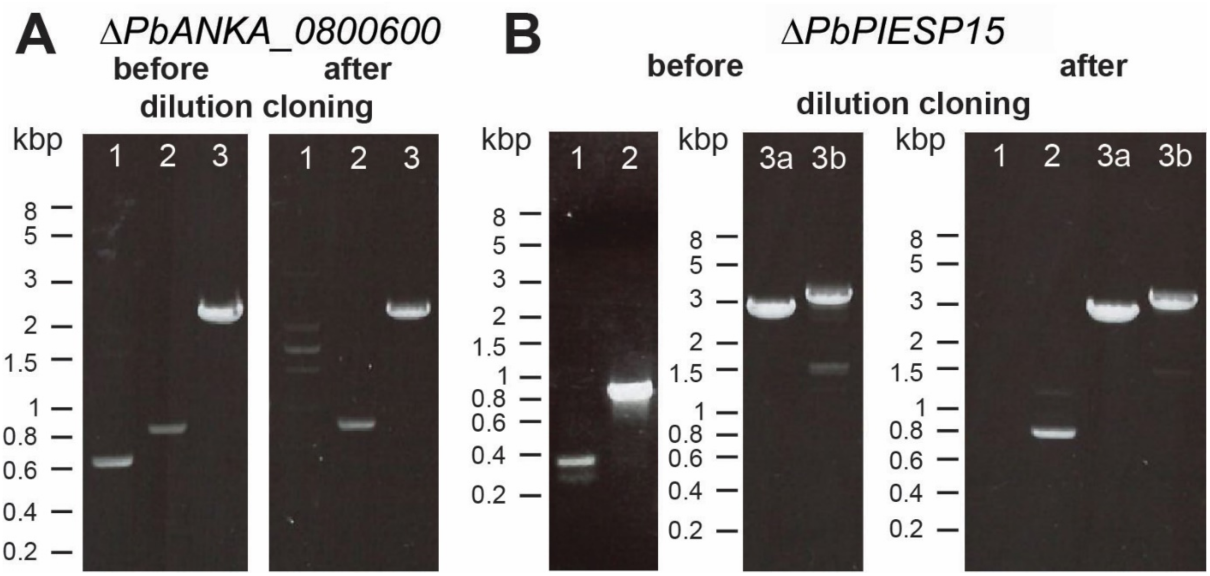

Figure S3. Genotyping of Δ*PbANKA_0800600* and Δ*PbPIESP15* parasites. Genomic DNA was isolated from Δ*PbANKA_0800600* (**A**) and Δ*PbPIESP15* (**B**) parasites before (left) and after (right) dilution cloning. Primers were designed to detect the WT locus (1), the transfection vector (2) and the integrated transfection cassette (3). After dilution cloning, the WT genes were not detected, demonstrating that the parasite lines are clonal and only contain recombinant parasites. Anticipated recombination at the *PIESP15* locus was confirmed with two PCR reactions, each amplifying one half of the required sequence (3a, 3b).

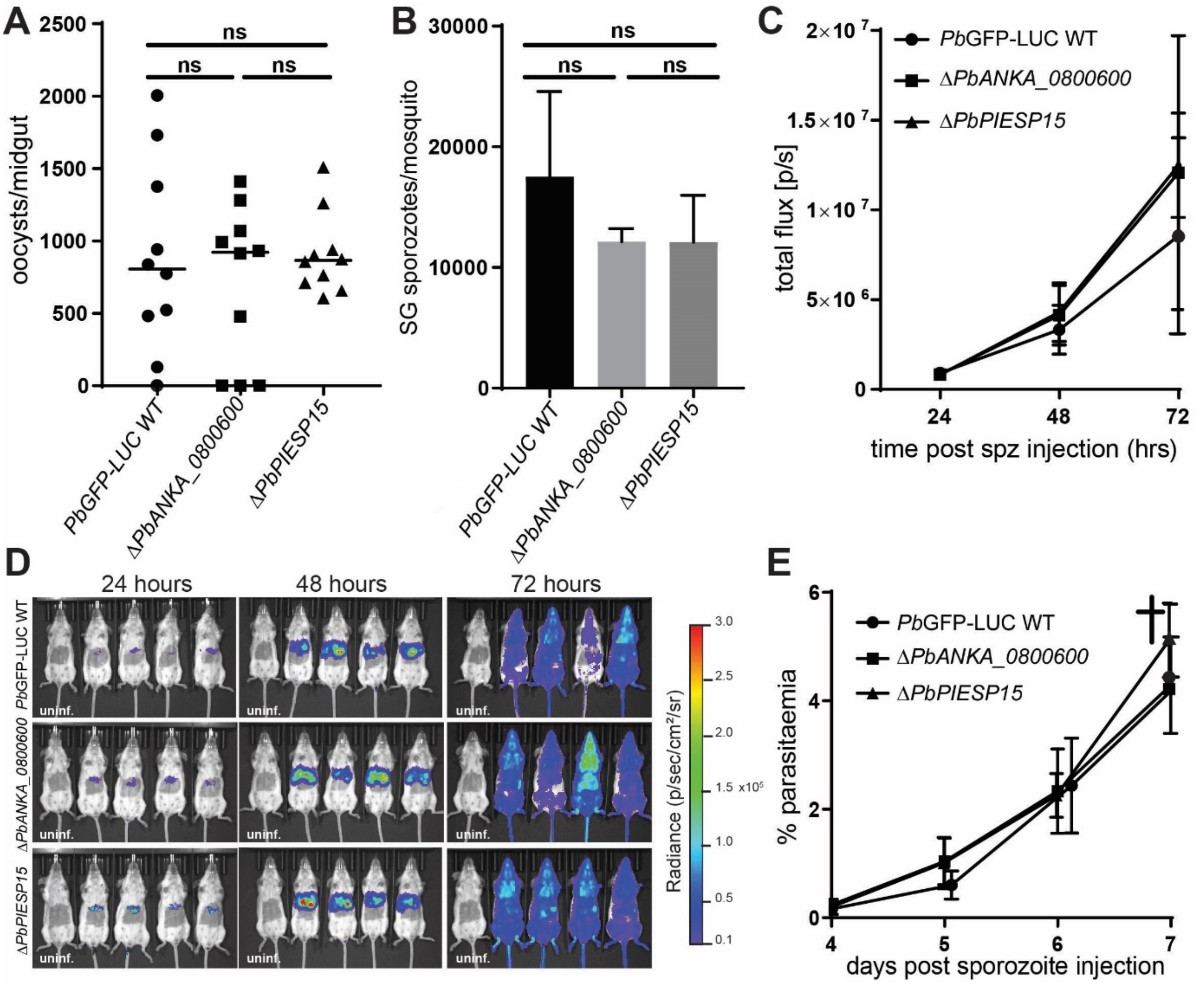

Figure S4. Δ*PbPIESP15* and Δ*PbANKA_0800600* parasites exhibit no overt infection phenotype. (**A**) The number of oocysts per midgut in *A. stephensi* mosquitoes infected with *P. berghei* genetically targeted for *PbPIESP15* and *PbANKA_0800600* were not significantly different from the GFP-luciferase transgenic wild-type parental strain. Data points are individual midguts; median is indicated and statistical comparisons were performed using the Mann-Whitney Test; P-values > 0.05 were considered non-significant (ns). (**B**) The average number of salivary gland (SG) sporozoites per mosquito is plotted. 15-20 mosquitoes were dissected and sporozoites isolated from the salivary glands were counted in a hemocytometer; columns represent the average number of sporozoites per mosquito taken from four independent experiments. Shown is the mean + SD (**C**) Δ*PbPIESP15* and Δ*PbANKA_0800600* parasites exhibit no overt infection phenotype compared to wild-type control. Groups of four mice were infected by intravenous administration of 5000 isolated salivary gland sporozoites and the resulting parasitaemia quantified using luciferase-based bioluminescence of the entire animal by an *in vivo* imaging system. Data points are mean ± SD; n = 4. (**D**) Normalised bioluminescence images collected on each day after infection; the left-most animal in each cohort is an uninfected control. (**E**) The asexual blood stage parasitaemia in Δ*PbPIESP15* and Δ*PbANKA_0800600* parasites was quantified by microscopic analysis of blood smears taken between day four and seven post infection and showed no difference compared to the wild-type control. All animals were culled on day 7 because they showed symptoms of disease (indicated by the cross) Shown are the results from one of three independent experiments with similar results. Data points are mean ± SD; n = 4.

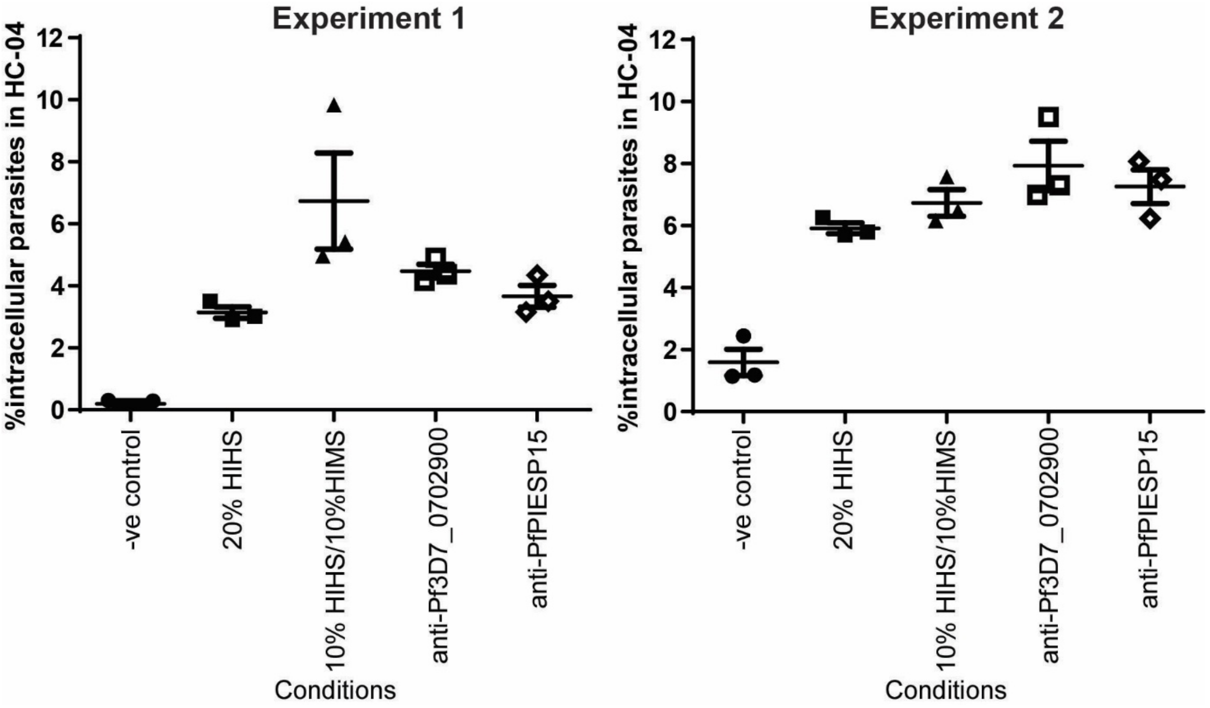

Figure S5. Polyclonal antisera elicited against Pf3D7_0702900 and PfPIESP15 do not affect *P. falciparum* NF-135 sporozoite invasion of the human HC-04 cell line. *P. falciparum* NF-135 sporozoites were incubated with either 20% heat inactivated human serum (HIHS) or a mixture of 10% human serum and 10% mouse serum (10% HIHS/10% HIMS), 10% anti-Pf3D7_0702900 or anti-PfPIESP15 polyclonal antiserum before being incubated with HC-04 cells for three hours. Cells incubated with media only (no sporozoites) were used as negative control. Invasion was quantified as percentage of intracellular parasites by FACS analysis of cells stained with an anti-CSP antibody. Data points show mean ± SEM, $n = 3$. Data from two independent experiments are shown and comparison of the results using a one-way ANOVA showed no significant difference between anti-Pf3D7_0702900 or anti-PfPIESP15 treated sporozoites and controls.

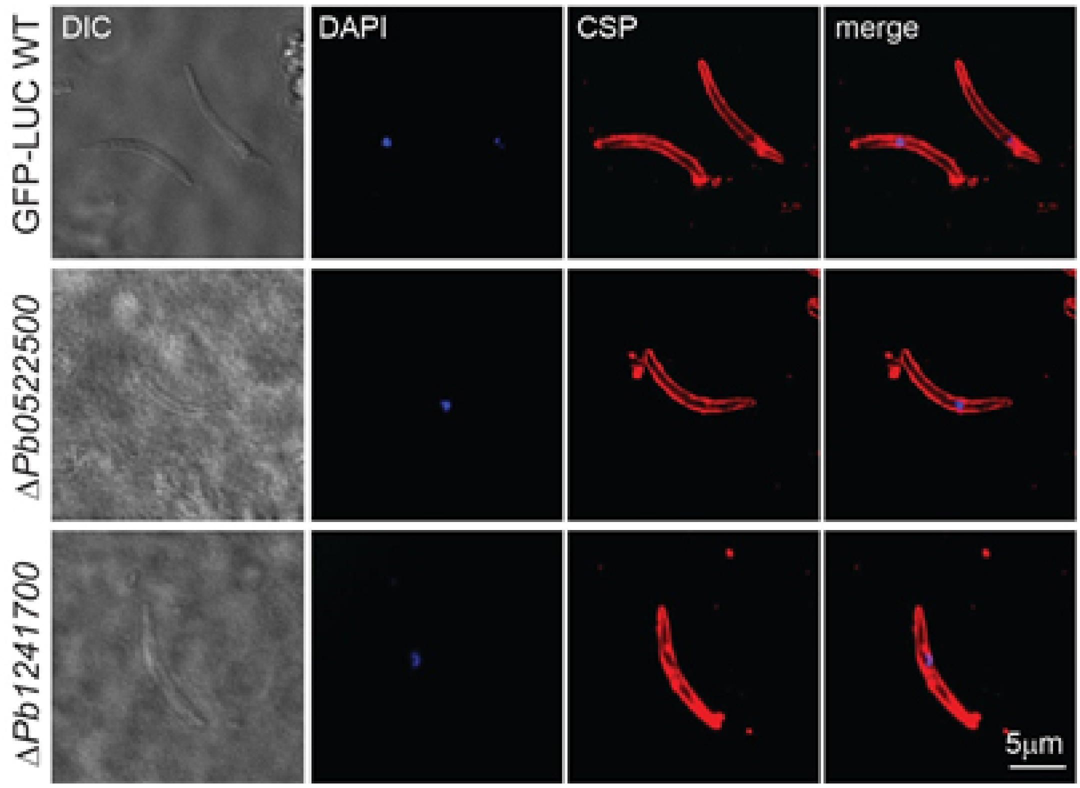

Figure S6. The amount and localisation of CSP protein does not differ between *P. berghei* GFP-LUC WT and Δ*Pb0522500* and Δ*Pb1241700* sporozoites. Air-dried and Triton-treated *P. berghei* GFP-LUC WT and Δ*Pb0522500* and Δ*Pb1241700* sporozoites were stained with a monoclonal antibody against the CSP protein. No overt difference could be observed in the staining pattern showing that the amounts and subcellular localisation of the GPI-anchored CSP protein is not affected in parasites lacking either the *Pb0522500* or *Pb1241700* gene.

Table S1. A library of sporozoite surface and secreted proteins. The protein sequences from the *P. falciparum* reference strain 3D7 were truncated to remove their endogenous signal peptides and transmembrane domain or GPI anchor if present. The region of the protein (typically the full length ectodomain) which was codon optimised and synthesised for expression is identified by the N- and C-terminal residues and their position. The constructs were transfected transiently into HEK293 cells using a non-commercial transfection agent (PEI) and expression levels are based on the recombinant proteins' β-lactamase activity or activity in ELISA (in the case of merozoite proteins, previously published). Key: n.d. = not detected; subcloning failed = insert failed to be cloned into expression plasmid; synthesis failed = gene synthesis repeatedly failed; n/a = insert not subcloned into bait expression vector

Localisation	no.	Addgene ID	Group	Localisation	Accession Number	Protein Description	Synonyms	Region Expressed	Expression level prey	Expression level bait
**Transmembrane**	1	110954	Ookinete	anchored component of plasma membrane (cell surface)	PF3D7_1346800	6-cysteine protein (P47)	PF47, PFS47	E27-T418	very low	n.d.
	2	110955	Midgut Sporozoite	Apicoplast	PF3D7_1213500	integral membrane protein GPR180, putative		K28-E430	n.d.	high
	3	110956	Midgut Sporozoite	ER, IMC, plama membrane	PF3D7_0918000	secreted acid phosphatase (GAP50)	PfGAP50, PfSAP, SAP	Q26-P360	high	high
	4		Midgut Sporozoite	anchored component of external side of plasma membrane	PF3D7_0718300	CRMP2	PfCRMP2	T28-M1735	subcloning failed	subcloning failed
	5		Midgut Sporozoite	anchored component of external side of plasma membrane	PF3D7_0911300	CRMP1	PfCRMP1	L11-M2387	subcloning failed	subcloning failed
	6	110957	Midgut Sporozoite	anchored component of plasma membrane	PF3D7_1346700	6-cysteine protein (P48/45)	PFS48/45	N28-S427	low	n.d.
	7	110958	Midgut Sporozoite	unknown	PF3D7_0103900	parasite-infected erythrocyte surface protein (PIESP15)		K25-K539	medium	low
	8	110959	Midgut Sporozoite	unknown	PF3D7_0910300	conserved Plasmodium protein, unknown function		E29-S368	medium	high
	9	110960	Midgut Sporozoite	unknown	PF3D7_1105300	conserved Plasmodium protein, unknown function		Y22-L678	n.d.	n.d.
	10	110961	Midgut and Salivary gland sporozoites	Apicoplast	PF3D7_1442600	TRAP-like protein,sporozoite-specific transmembrane protein S6 (TREP)	S6, UOS3	M1-P384	n.d.	low
	11	110962	Midgut and Salivary gland sporozoites	Rhoptries	PF3D7_1452000	rhoptry neck protein 2 (RON2)	PFRON2	S21-V2072	n.d.	n.d.
	12	110963	Midgut and Salivary gland sporozoites	Micronemes	PF3D7_1147800.1	merozoite adhesive erythrocytic binding protein (MAEBL)	PFMAEBL	I21-N1987	n.d.	n.d.
	13	110964	Midgut and Salivary gland sporozoites	Cell surface	PF3D7_0104000	thrombospondin related sporozoite protein (TRSP)	sporozoite stage TSP1 domain protein	D20-T134	low	medium
	14	110965	Midgut and Salivary gland sporozoites	Host cell cytosol	PF3D7_1463900	EF-hand calcium binding domain containing protein, putative		N22-A447	very low	low
	15	110966	Midgut and Salivary gland sporozoites	unknown	PF3D7_0912400	alkaline phosphatase, putative		Q21-T403	n.d.	n.d.
	16		Midgut and Salivary gland sporozoites	unknown	PF3D7_1229300	conserved Plasmodium protein		L20-S890	synthesis failed	n/a
	17	110967	Midgut and Salivary gland sporozoites	unknown	PF3D7_1310500	conserved Plasmodium protein		D30-P147	very low	high
	18	110968	Midgut and Salivary gland sporozoites	unknown	PF3D7_1350600	conserved Plasmodium protein		M22-T196	low	n.d.
	19	110969	Midgut and Salivary gland sporozoites	unknown	PF3D7_1352500	thioredoxin-related protein, putative		Q23-P170	low	high
	20	110970	Midgut and Salivary gland sporozoites	unknown	PF3D7_1462300	conserved Plasmodium protein		I19-P1149	n.d.	low
	21	110971	Salivary gland sporozoites	Apicoplast	PF3D7_0902900	conserved Plasmodium protein		S24-D54	medium	high
	22	110972	Salivary gland sporozoites	Apicoplast	PF3D7_1333300	transmembrane protein Tmp21 homologue, putative		Y18-A178	medium	low
	23	110973	Salivary gland sporozoites	Rhoptries	PF3D7_0511600	apical rhoptry neck protein (ARNP)		F20-N179	low	high
	24	110974	Salivary gland sporozoites	Cell surface	PF3D7_0511400	conserved Plasmodium protein		S25-T330	low	medium
	25	110975	Salivary gland sporozoites	Cell surface	PF3D7_0812300	sporozoite surface protein 3 (SSP3)		E23-P411	n.d.	medium
	26	110976	Salivary gland sporozoites	Integral component of membrane, symbiont containing vacuole	PF3D7_0406200	sexual stage-specific protein precursor (Pfs16)		D26-R103	high	medium
	27	110977	Salivary gland sporozoites	Symbiont-containing vacuole membrane	PF3D7_0829600	early transcribed membrane protein 8 (ETRAMP8)	ETRAMP.BLOB.2	K25-K52	high	low
	28	110978	Salivary gland sporozoites	Symbiont-containing vacuole membrane	PF3D7_1016900	early transcribed membrane protein 10.3 (ETRAMP10.3)	PEG4, UIS4	L25-Q52	high	high
	29	110979	Salivary gland sporozoites	Maurer's cleft	PF3D7_1201300	liver stage associated protein 1 (LSAP1)	ETRAMP, LSAP-1	V25-T52	high	high
	30	110980	Salivary gland sporozoites	unknown	PF3D7_0929900	conserved Plasmodium protein		Y21-S139	low	n.d.
	31	110981	Salivary gland sporozoites	unknown	PF3D7_1434400	conserved Plasmodium membrane protein		N23-N265	low	medium
	32	110982	Salivary gland sporozoites	unknown	PF3D7_0105400.1	conserved Plasmodium protein		K21-S85	medium	medium
	33	110983	Salivary gland sporozoites	unknown	PF3D7_0108700	secreted ookinete protein (PSOP24)		K20-P456	medium	medium
	34	110984	Salivary gland sporozoites	unknown	PF3D7_0422100	transmembrane emp24 domain-containing protein, putative		N23-T350	medium	medium
	35	110985	Salivary gland sporozoites	unknown	PF3D7_0526900	transmembrane emp24 domain-containing protein, putative		A28-N180	medium	high
	36	110986	Salivary gland sporozoites	unknown	PF3D7_0713700	conserved Plasmodium protein		D22-P166	high	high
	37	110987	Salivary gland sporozoites	unknown	PF3D7_1123500	conserved Plasmodium protein		N24-T1222	n.d.	low
	38	110988	Salivary gland sporozoites	unknown	PF3D7_0505700	conserved Plasmodium membrane protein		E22-T515	medium	low
**GPI**	39	110989	Ookinete	Micronemes	PF3D7_0801300	von Willebrand factor A-domain related protein (WARP)		R24-S290 (S mutated to A)	low	medium
	40	110990	Ookinete	Membrane	PF3D7_1030900	ookinete surface protein P28	PFS28	R23-S194	high	high
	41	110991	Ookinete	Anchored component of plasma membrane	PF3D7_1031000	ookinete surface protein P25	PFS25	K23-T193	high	high
	42	110992	Midgut and Salivary gland sporozoites	Anchored component of plasma membrane, host cell cytoplasm, host cell nucleus	PF3D7_0304600	circumsporozoite (CS) protein (CSP)	CS, PfCSP	M1-S376	medium	medium
	43	110993	Midgut and Salivary gland sporozoites	Cell surface	PF3D7_0620000	secreted ookinete protein 25, putative	POS8	K25-S485	medium	Clone not available at time of screening, clone available now
**secreted**	44	110994	Ookinete	Micronemes	PF3D7_0315200	circumsporozoite- and TRAP-related protein (CTRP)		H24-N2114	n.d.	n.d.
	45	110995	Ookinete	Micronemes	PF3D7_1252200	chitinase (CHT1)	PFCHT1	H29-H378	n.d.	low
	46	110996	Ookinete	Micronemes	PF3D7_1340000	secreted ookinete protein, putative (PSOP7)		L23-F618	n.d.	low
	47	110997	Ookinete	Micronemes	PF3D7_1404300	secreted ookinete adhesive protein (SOAP)		K20-C202	medium	high
	48	110998	Ookinete	Crystalloid, symbiont containing vacuole	PF3D7_1455800	LCCL domain-containing protein (CCp2)	LAP4	G24-P1617	n.d.	n.d.
	49	110999	Ookinete	Crystalloid, symbiont containing vacuole	PF3D7_1475500	LCCL domain-containing protein (CCp1)	LAP2	Q20-I1620	n.d.	n/a (not expressed for AVEXIS)

	50		Midgut Sporozoite	Apicoplast	PF3D7_1367800	secreted ookinete protein, putative (PSOP2)		E23-I1182	subcloning failed	subcloning failed
	51	111000	Midgut Sporozoite	Cell surface	PF3D7_0513700	secreted ookinete protein, putative (PSOP12)		F24-I735	n.d.	low
	52	111001	Midgut Sporozoite	Cytosol, food vacuole	PF3D7_1454400	aminopeptidase P (APP)	PFAPP	K23-N777	low	medium
	53	111002	Midgut and Salivary gland sporozoites	Apical part of cell	PF3D7_0408600	sporozoite invasion associated protein 1 (SIAP-1)	AG17, PfSIAP-1, S5	Y22-D984	n.d.	low
	54	111003	Midgut and Salivary gland sporozoites	Apicoplast	PF3D7_0721700	secreted ookinete protein (PSOP1)		V17-P467	high	high
	55	111004	Midgut and Salivary gland sporozoites	Apicoplast	PF3D7_1232100	CPN60		I24-E718	medium	low
	56	111005	Midgut and Salivary gland sporozoites	Microneme	PF3D7_1335900	thrombospondin-related anonymous protein (TRAP)	SSP2	M1-P497	high	high
	57	111006	Midgut and Salivary gland sporozoites	Microneme	PF3D7_1342500	sporozoite protein essential for cell traversal (SPECT1)	PFSPECT, SPECT	Y22-S245	medium	high
	58		Midgut and Salivary gland sporozoites	Rhoptries	PF3D7_1116000	rhoptry neck protein 4 (RON4)	PFRON4	F24-L1201	synthesis failed	n/a
	59	111007	Midgut and Salivary gland sporozoites	ER	PF3D7_1115600	peptidyl-prolyl cis-trans isomerase (CYP19B)	PFCYP19B	A22-L195	low	medium
	60	111008	Midgut and Salivary gland sporozoites	Cell surface, ER	PF3D7_1222300	endoplasmin, putative (GRP94)	HSP90	D29-L821	low	medium
	61	111009	Midgut and Salivary gland sporozoites	unknown	PF3D7_1351800.1	conserved Plasmodium protein		V20-S230	high	high
	62	111010	Salivary gland sporozoites	Apicoplast, cell periphery	PF3D7_0721100	conserved Plasmodium protein		R25-E244	n.d.	high
	63	111011	Salivary gland sporozoites	Apicoplast	PF3D7_0729200	1-cys peroxiredoxin (AOP)	PfSOP, PRX	F21-L240	medium	medium
	64	111012	Salivary gland sporozoites	Apicoplast	PF3D7_1106200	conserved Plasmodium protein		N17-N681	n.d.	low
	65	111013	Salivary gland sporozoites	Apicoplast	PF3D7_1234600	protein TOC75, putative		K24-L1130	n.d.	n/a
	66	111014	Salivary gland sporozoites	Micronemes	PF3D7_1216600	cell traversal protein for ookinetes and sporozoites (CelTOS)		F25-D182	high	high
	67	111015	Salivary gland sporozoites	Micronemes, rhoptry neck	PF3D7_1449000	gamete egress and sporozoite traversal protein (GEST)		I21-Q248	low	low
	68	111016	Salivary gland sporozoites	Rhoptries	PF3D7_0722200	rhoptry associated leucine zipper-like protein 1 (RALP1)		S23-F749	n.d.	low
	69	111017	Salivary gland sporozoites	Cell surface, rhoptries	PF3D7_1012200	rhoptry associated adhesin		E18-K267	low	low
	70	111018	Salivary gland sporozoites	ER	PF3D7_1404900	conserved Plasmodium protein		R24-L297	high	high
	71	111019	Salivary gland sporozoites	Food vacuole, symbiont-containing vacuole	PF3D7_1116700	cathepsin C, homolog,dipeptidyl aminopeptidase 1 (DPAP1)		D28-N700	n.d.	medium
	72	111020	Salivary gland sporozoites	Plasma membrane	PF3D7_0317100	6-cysteine protein (B9)		G22-F969	medium	low
	73	111021	Salivary gland sporozoites	unknown	PF3D7_0107300	conserved Plasmodium protein		I23-K399	n.d.	low
	74	111022	Salivary gland sporozoites	unknown	PF3D7_0624400	conserved Plasmdium protein		D20-K607	low	low
	75	111023	Salivary gland sporozoites	unknown	PF3D7_0702900	conserved Plasmdium protein		Q24-N107	high	high
	76	111024	Salivary gland sporozoites	unknown	PF3D7_0909200	GDP fucose protein O-fucosyltransferase 2 (POFUT2)		V19-T469	n.d.	n.d.
	77	111025	Salivary gland sporozoites	unknown	PF3D7_1242000	conserved Plasmodium protein		D28-S531	very low	low

previously published proteins:

**transmembrane**	78		Ookinete	Rhoptries	PF3D7_0501500	rhoptry-associated protein 3 (RAP3)		N23-K400	low	n.d.
	79		Midgut Sporozoite	unknown	PF3D7_1334600	MSP7-like protein (MSRP3)		Q24-S298	low	medium
	80		Midgut and Salivary gland sporozoites	Micronemes, rhoptry neck	PF3D7_1133400	apical membrane antigen 1 (AMA1)	PF83, PFAMA1, RMA1	Q25-T541	high	high
	81		Midgut and Salivary gland sporozoites	Micronemes, rhoptry neck	PF3D7_0405900	apical sushi protein (ASP)	RON1	A20-S708	low	high
	82		Midgut and Salivary gland sporozoites	Rhoptries	PF3D7_1017100	rhoptry neck protein 12 (RON12)		V22-E310	high	high
	83		Midgut and Salivary gland sporozoites	Rhoptries	PF3D7_1252100	rhoptry neck protein 3 (RON3)		N22-N249	n.d.	n/a
	84		Midgut and Salivary gland sporozoites	Anchored component of plasma membrane	PF3D7_0612800	6-cysteine protein (P12p)		Y21-T349	low	low
	85		Midgut and Salivary gland sporozoites	Integral component of membrane	PF3D7_0616500	TRAP-like protein (TLP)	TRAP2	E24-P1306	low	high
	86		Salivary gland sporozoites	Food vacuole, plasma membrane, symbiont-containing vacuole	PF3D7_0502400	ring-stage membrane protein 1,merozoite surface protein 8 (MSP8)	RMP1	E26-S576	medium	high
**GPI**	87		Midgut Sporozoite	Anchored component of plasma membrane (cell surface)	PF3D7_1364100	6-cysteine protein (P92),	PF92	N27-S770	n.d.	low
	88		Midgut and Salivary gland sporozoites	Anchored component of plasma membrane, cell surface, micronemes	PF3D7_0828800	GPI-anchored micronemal antigen (GAMA)	PSOP9	L22-P710	medium	high
	89		Midgut and Salivary gland sporozoites	Rhoptries	PF3D7_0419700	apical merozoite protein (Pf34)	PV2	N25-S306	medium	high
	90		Salivary gland sporozoites	Anchored component of plasma membrane, cell surface, microneme	PF3D7_0508000	6-cysteine protein (P38)	Pf38, Pfs38	Q22-S328	low	medium
	91		Salivary gland sporozoites	Anchored component of plasma membrane	PF3D7_0930300	merozoite surface protein 1 (MSP1)	MSA1, Pf190, Pf195, PMMSA	V20-S1701	high	high
	92		Salivary gland sporozoites	Rhoptries	PF3D7_0707300	rhoptry-associated membrane antigen (RAMA)		Y17-K838	medium	low
**secreted**	93		Ookinete	unknown	PF3D7_1335000	MSP7-like protein (MSRP1)		Y22-T379	high	low
	94		Midgut and Salivary gland sporozoites	Rhoptries	PF3D7_0214900	rhoptry neck protein 6 (RON6)		F16-T949	low	low
	95		Midgut and Salivary gland sporozoites	Rhoptries	PF3D7_0501600	rhoptry-associated protein 2 (RAP2)		D22-L398	n.d.	n/a
	96		Midgut and Salivary gland sporozoites	Rhoptries	PF3D7_0905400	high molecular weight rhoptry protein 3 (RhopH3)		K25-L897	low	medium
	97		Salivary gland sporozoites	Cell surface, rhoptries, micronemes	PF3D7_0212600	secreted protein with altered thrombospondin repeat domain (SPATR)		E22-C250	high	high
	98		Salivary gland sporozoites	Rhoptries	PF3D7_0302200	cytoadherence linked asexual protein 3.2 (CLAG3.2)	RhopH1	K21-H1416	n.d.	subcloning failed
	99		Salivary gland sporozoites	Rhoptries	PF3D7_0929400	high molecular weight rhoptry protein 2 (RhopH2)		L20-S1378	n.d.	n.d.
	100		Salivary gland sporozoites	Rhoptries	PF3D7_1410400	rhoptry-associated protein 1 (RAP1)	PFRAP-1, PFRAP1, RAP-1	I23-D728	n.d.	low
	101		Salivary gland sporozoites	Anchored component of plasma membrane (cell surface)	PF3D7_0404900	6-cysteine protein (P41)	Pf41, Pfs41	K21-S378	high	high
	102		Salivary gland sporozoites	unknown	PF3D7_1404700	conserved Plasmodium protein		Q21-K290	low	low

Table S2. Primer sequences for cloning and genotyping

Primer name	sequence
**cloning:**	
Pb 0522500-3UTR-Xho-F	agaCTCGAGGAATTGCGGCCGCACCATTTATATTATATCCACA
Pb 0522500-3UTR-Sac-R	agaCCGCGGCATCATACAAATAAACTTGG
Pb 0522500-5UTR-Kpn-F	agaGGTACCGGGCACACCAACTGACGCGCACGAATTACAATACCTCGAAAATATGTATTC
Pb 0522500-5UTR-Xma-R	agaCCCGGGGCGGCCGCTTTGATAGAATGCATTGCTAGCTA
Pb 1241700-3UTR-Xho-F	agaCTCGAGGAATTGCGGCCGCAGATCATACTTTTGTGAACATTTTTGTT
Pb 1241700-3UTR-Sac-R	agaCCGCGGAAAGGAATAATATTGATTGGATATTTA
Pb 1241700-5UTR-Kpn-F	agaGGTACCATAAGCTTTTCTGACGCGCACGAATTACTCACAACTTTCATAATAAAAAAATTATTAAG
Pb 1241700-5UTR-Xma-R	agaCCCGGGGCGGCCGCGATATAATTTTATGTTTCTGTTTTTTTTTAGTATG
**genotyping:**	
Pb PIESP15-fwd2	GGTTACATAAAGTCAGGAT
Pb PIESP15-rev2	GAATCCTGACTTTATGTAAC
Pb PIESP15-QCR1	TTTGTTGACTCCCCCTCACA*
Pb PIESP15-QCR2	TTTCCAAAGCTTATGCGGCA*
Pb PIESP15-GT	AGGCTACTCGAAAATGGCAAGCA*
Pb ANKA_0800600-QCR1	TCCTCTGCGTCAATTAAGCCT*
Pb ANKA_0800600-QCR2	GCAAGCGCCCTCTTATTTATGT*
Pb ANKA_0800600-GT	TGTTCCTTTGCGTCATGGTT*
Pb 0522500-QCR1	TTGTATATACAATTTCACC
Pb 0522500-QCR2	TGAAGCTATTAACGATGATAG
Pb 0522500-GT	GTTAGCATCACTTAAGATAG
Pb 1241700-QCR1	TATAGCATTTTAATCTTTA
Pb 1241700-QCR2	TTCCACATATAAGAACGAGTG
Pb 1241700-GT	GAGCTGGCAGCATATTTGTC
Universal GW2a	CGACAGAGGTCTAGATG
Universal GW2b	CAAGTATCTAGTTCCAGG

* = primer sequences were obtained from the PlasmoGEM web page
